# Environmental enrichment ameliorates perinatal brain injury and promotes functional white matter recovery

**DOI:** 10.1038/s41467-020-14762-7

**Published:** 2020-02-19

**Authors:** Thomas A. Forbes, Evan Z. Goldstein, Jeffrey L. Dupree, Beata Jablonska, Joseph Scafidi, Katrina L. Adams, Yuka Imamura, Kazue Hashimoto-Torii, Vittorio Gallo

**Affiliations:** 1 Center for Neuroscience Research, Children’s Research Institute, Children’s National Hospital, Washington, DC 20010 USA; 2 Institute for Biomedical Sciences, The George Washington University, Washington, DC 20052 USA; 3 Department of Anatomy and Neurobiology, Virginia Commonwealth University, Richmond, VA 23284 USA; 4 Institute for Personalized Medicine, Penn State University, College of Medicine, Hershey, PA 17033 USA

**Keywords:** Neuroscience, Development of the nervous system, Glial biology, Gliogenesis, Myelin biology and repair

## Abstract

Hypoxic damage to the developing brain due to preterm birth causes many anatomical changes, including damage to the periventricular white matter. This results in the loss of glial cells, significant disruptions in myelination, and thereby cognitive and behavioral disabilities seen throughout life. Encouragingly, these neurological morbidities can be improved by environmental factors; however, the underlying cellular mechanisms remain unknown. We found that early and continuous environmental enrichment selectively enhances endogenous repair of the developing white matter by promoting oligodendroglial maturation, myelination, and functional recovery after perinatal brain injury. These effects require increased exposure to socialization, physical activity, and cognitive enhancement of surroundings—a complete enriched environment. Using RNA-sequencing, we identified oligodendroglial-specific responses to hypoxic brain injury, and uncovered molecular mechanisms involved in enrichment-induced recovery. Together, these results indicate that myelin plasticity induced by modulation of the neonatal environment can be targeted as a therapeutic strategy for preterm birth.

## Abstract (web summary)

Hypoxic brain damage associated with premature birth causes lasting neurological impairments. Here, the authors use environmental enrichment to rescue white matter dysmaturation following hypoxia, while identifying a critical window of intervention and oligodendrocyte-specific changes in gene expression.

## Introduction

Perinatal hypoxia (HX) is a significant cause of preterm brain injury^1,2^. This oxygenation failure predisposes premature infants to diffuse white matter (WM) injury—a debilitating condition involving maturational delays in the oligodendrocyte (OL) population. OLs, the myelin-forming glia that ensheath axons in the central nervous system (CNS), are highly susceptible to HX-induced oxidative stress in both humans and rodents^2,3^. Following HX, OLs fail to fully mature, leading to persistent aberrations in myelin ultrastructure that are associated with morbid neurodevelopmental impairment and permanent disability^4–8^. Despite ever-rising rates of preterm brain injury, there remains no viable approach to attenuate the impact of perinatal HX on developing WM. Therefore, deeper understanding of OL injury and recovery after HX is essential, and may provide new approaches for functional recovery in the developing preterm infant.

Due to the plasticity of the developing brain, recovery from diffuse WM injury is primed for influence from exogenous environmental stimuli. Indeed, the environment in which premature infants are reared has profound effects on cognitive and behavioral outcomes^9^. Previous studies demonstrate that the environment affects neural plasticity and functional recovery after brain injury^10–12^. Further, environmental stimulation promotes structural changes in myelin, resulting in the modulation of neural circuits and neurological function^13–15^. In early life, this myelin plasticity evokes reorganization and remodeling of brain circuitry that could reduce neurodevelopmental disability in children born preterm^16^. However, significant challenges limit the investigation of the human brain, highlighting the necessity for experimentation on preclinical animal models.

Conveniently, the divergent neurodevelopmental timeline of rodents and humans allows for recapitulation of diffuse WM injury in the infant brain using a mouse model of chronic perinatal HX^8,17,18^. Using this model, we utilized environmental enrichment (EE) to elucidate the influence of exogenous input on WM development and recovery during critical neurodevelopmental periods. EE refers to a complex domain that emulates the natural habitat and challenges an animal to continuously adapt to its surroundings through exposure to novel stimuli, greater social interactions, and voluntary physical activity^19–21^. Recent studies highlight the utility of enrichment-based intervention after adult injury and the pivotal role of OLs in the neuromodulatory response to environmental experience^22–26^.

Here, we determine—for the first time—the developmental effects of EE on normal and injured WM, and provide a much-needed bridge between the dynamics of OL plasticity and WM-associated behavioral recovery. Early and continuous EE-intervention after HX enhanced oligodendrogenesis and myelination, resulting in improved behavioral performance. Further, we find that enrichment-induced de novo OL generation is required for functional recovery, indicating that the environment plays a substantial role in the formation of new myelin after injury. Finally, using translating ribosome affinity purification (TRAP) and RNA-seq, we detail the dynamic transcriptional changes occurring in the OL-lineage after developmental brain injury, and begin to elucidate the complex and multifaceted molecular mechanisms underlying enrichment-induced recovery in glia. While perinatal HX delays the developmental trajectory of oligodendroglia, our novel findings indicate that EE accelerates the endogenous oligodendrogenic response to injury, and induces additional changes in genes that promote myelination. Collectively, this suggests that EE affects the entire developmental program of OLs by exerting important changes on different families of genes at different critical windows in response to injury. Likely, this age-specific environmental manipulation of gene networks resynchronizes glial and neuronal developmental programs to promote functional myelination and ultimately, confer functional recovery. Further investigation into specific candidate pathways and gene networks known to influence recovery will inform the development of new therapeutic strategies aimed at harnessing endogenous mechanisms of repair.

## Results

### EE promotes OL maturation and myelination after hypoxia

**Fig. 1:**
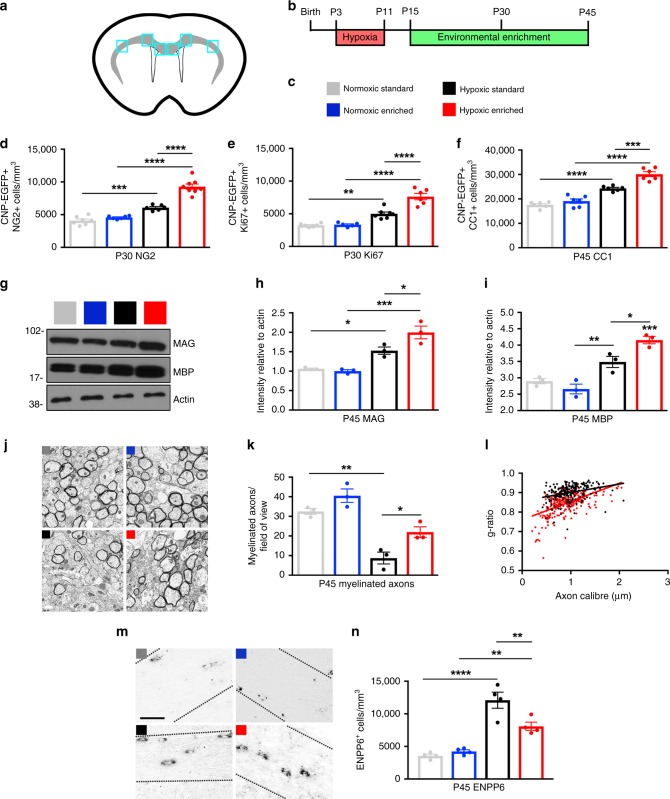
Environmental enrichment promotes oligodendroglial maturation and myelination after perinatal brain injury. **a** Schematic of the subcortical white matter (gray) in a coronal slice, with boxes representing the quantified regions (corpus callosum, cingulum, external capsule). **b** Experimental paradigm. **c** Experimental groups. **d** Quantification of EGFP + NG2 + OPCs at P30 (NX Standard (NX), *n* = 7; NX Enriched (NX-EE), *n* = 6; HX Standard (HX), *n* = 6; HX Enriched (HX-EE), *n* = 8) (***p = 0.0003; ****p < 0.0001). **e** Quantification of EGFP + Ki67 + proliferating OPCs at P30 (NX, *n* = 6; NX-EE, *n* = 5; HX, *n* = 7; HX-EE, *n* = 6) (**p = 0.002; ****p < 0.0001). **f** Quantification of EGFP + CC1 + OLs at P45 (*n* = 6 per group) (***p = 0.0001; ****p < 0.0001). **g** Representative western blot for myelin proteins MAG and MBP in the subcortical white matter of the four experimental groups at P45 (*n* = 3 per group). **h** Quantification of MAG protein levels, normalized to Actin (from left to right: *p = 0.038; ***p = 0.0004; *p = 0.037). **i** Quantification of MBP protein levels, normalized to Actin (*p = 0.03; **p = 0.009; ***p = 0.0007). **j** Representative EM images from P45 white matter. Experiments were performed once using CNP-EGFP mice. Scale bar = 0.5 μm. **k** Quantification of myelinated axon number in the corpus callosum (*n* = 3 per group) (*p = 0.039, **p = 0.001). **l** Scatter plot of calculated g-ratios of individual corpus callosum axons relative to axon diameters in HX vs. HX-EE. **m** Representative images of ENPP6 in situ hybridizations from P45 white matter. Experiments were performed once using CNP-EGFP mice. Scale bar = 50 μm. **n** Quantification of high ENPP6-expressing cells at P45 (*n* = 4 per group) (from left to right: ****p < 0.0001, **p = 0.003, **p = 0.008). Unless otherwise noted, data expressed as mean ± SEM. One-way ANOVA followed by a Tukey’s post hoc test for multiple comparisons was used to compare differences between groups. Source data are provided as a Source Data file.

EE promotes the recovery of inhibitory interneurons after HX-induced developmental delay^18^, and enhances the generation of OL progenitors (OPCs) after focal cortical ischemia in the adult brain^26,27^. However, the effects of EE on OL development following perinatal HX remain unknown. Therefore, we characterized OL dynamics in the subcortical WM (Fig. 1a) following HX-induced WM injury and EE-driven recovery. 2′,3′-cyclic nucleotide 3′-phosphodiesterase (CNP) enhanced green fluorescent protein (EGFP) mice—a widely used tool in which all OL lineage cells are fluorescently labeled^28,29^—were separated into four experimental groups: (1) Normoxic Standard (NX), which were reared in a standard laboratory environment; (2) Normoxic Enriched (NX-EE), which were placed into EE at P15, where they remained for the duration of the experiment; (3) Hypoxic Standard (HX), which were exposed to HX from P3 to P11 (10.5 ± 0.5% oxygen), and raised thereafter in a standard environment; and (4) Hypoxic Enriched (HX-EE), which were exposed to HX from P3 to P11, and then reared continuously in an enriched environment (Fig. 1b, c).

We first assessed the effects of HX on EGFP^+^NG2^+^-OPCs and EGFP^+^Ki67^+^-proliferating OPCs vs. NX controls, and found an increase in both cell types at P30 (HX vs. NX—OPCs: 6080 ± 167 cells/mm^3^ vs. 4090 ± 241; proliferating OPCs: 5006 ± 279 vs. 3243 ± 110) (Fig. 1d–e; Supplementary Fig. 1a–f). Interestingly, EE enhanced this endogenous response and significantly increased OPCs and proliferating OPCs after HX exposure (HX-EE vs. HX—OPCs: 9284 ± 393 cells/mm^3^ vs. 6080 ± 167; proliferating OPCs: 7632 ± 486 vs. 5006 ± 279), but had no effect on NX mice (NX-EE vs. NX—OPCs: 4576 ± 91 cells/mm^3^ vs. 4090 ± 241; proliferating OPCs: 3325 ± 137 vs. 3243 ± 110).

Because of the significant increase in OPCs after HX and subsequent EE, we next investigated corresponding changes in differentiated OLs at a later time point. There was a significant increase in the total number of EGFP^+^CC1^+^ OLs in the HX-EE group at P45 compared to HX controls (HX-EE vs. HX—OLs: 30,115 ± 1011 cells/mm^3^ vs. 24,302 ± 411) (Fig. 1f; Supplementary Fig. 1g, h), highlighting a robust OL response to EE following injury. There was also a significant increase in CC1^+^ OLs in the HX group vs. NX controls (HX vs. NX—OLs: 24,302 ± 411 cells/mm^3^ vs. 17,580 ± 492). This aligns with previous studies that reported an initial loss of OLs in the WM recovers approximately one month following HX injury^8,17^. Interestingly, at P22, P30, and P45, there were no differences in WM cells that expressed cleaved caspase-3, a marker of apoptosis (Supplementary Fig. 2), suggesting that the accumulation of OLs after EE-induced recovery from HX is a result of increased oligodendrogenesis, and not due to enhanced OL survival.

To further investigate the effects of EE under NX and HX conditions, we analyzed myelin protein expression in the subcortical WM. Consistent with an increase in OLs, EE significantly increased myelin proteins MAG and MBP after HX at P45 (*p* = 0.037 and *p* = 0.031, respectively; one-way ANOVA and Tukey’s post hoc), but not NX (Fig. 1g–i). To evaluate myelination at the ultrastructural level, we performed electron microscopy (EM) analysis on the corpus callosum of P45 mice. As predicted, recovery from HX in EE led to a significant increase in myelinated axons compared to HX controls (22 ± 2.7 axons/field of view vs. 8.7 ± 3.0) (Fig. 1j, k). Also, *g*-ratios were reduced in the HX-EE group compared to HX (Fig. 1i), indicative of thicker myelin. Interestingly, despite elevated OL and myelin protein levels, HX mice had dramatically fewer myelinated axons compared with NX (8.7 ± 3.0 axons/field of view vs. 32.5 ± 1.5), suggesting that many of the EGFP^+^CC1^+^ cells in the HX group are premyelinating OLs, which express MAG and MBP^15,30^. To verify this, we utilized in situ hybridization for ENPP6, which is highly expressed in premyelinating OLs^15^. Indeed, there was a significant increase in ENPP6^+^ cells in the WM of HX mice compared to NX (12,090 ± 1230 cells/mm^3^ vs. 3559 ± 244) (Fig. 1m, n). Although levels of ENPP6^+^ cells were still elevated in HX-EE vs. NX (8095 ± 628 cells/mm^3^ vs. 3559 ± 244), there were significantly less than HX controls (8095 ± 628 cells/mm^3^ vs. 12,090 ± 1230).

Lastly, to investigate if other cell types in the WM influence the EE-induced OL response following HX, immunohistochemical analyses were performed for axonal, astrocytic, and microglial markers (Supplementary Fig. 3). Interestingly, HX induced a decrease in the density of phosphorylated neurofilaments at P22 and P30 (HX vs. NX: P22—4.23 ± 0.87 %SMI31^+^ area vs. 11.08 ± 0.61; P30—4.03 ± 0.16 vs. 7.72 ± 0.23), which was partially rescued by EE (HX-EE vs. HX: P22—7.45 ± 0.83 %SMI31^+^ area vs. 4.23 ± 0.87; P30—5.98 ± 0.15 vs. 4.03 ± 0.16; Supplementary Fig. 3c–e). Conversely, the density of nonphosphorylated neurofilaments was increased after HX at P22 and P30 (HX vs. NX: P22—10.29 ± 1.07 %SMI32^+^ area vs. 4.29 ± 0.36; P30—6.34 ± 0.48 vs. 3.25 ± 0.06), which was partially rescued by EE at P22 (7.58 ± 0.15 %SMI32^+^ area vs. 10.29 ± 1.07), and fully rescued at P30 (4.01 ± 0.15 %SMI32^+^ area vs. 6.34 ± 0.48; Supplementary Fig. 3c, f, g). These data indicate that EE helps to reverse abnormal neurofilament phosphorylation, which is a common pathogenesis of many neurological disorders^31^. As for the other major cell populations in the WM, there were no differences at P22 or P30 in the density of astrocytic marker, GFAP, or microglial markers, IBA1, and CD68 (Supplementary Fig. 3h–m). This is in line with previous data showing no changes in astrocytes or microglia at earlier times following HX^17^. Furthermore, this indicates that WM astrocytes and microglia are unlikely to be directly involved in EE-induced OL maturation and myelination following perinatal brain injury.

### EE selectively enhances oligodendrogenesis after hypoxia

**Fig. 2:**
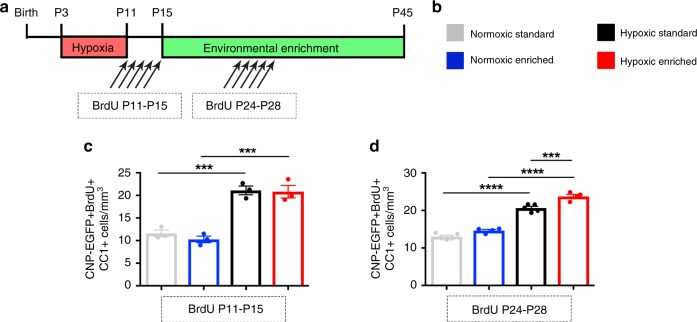
Environmental enrichment promotes oligodendrogenesis after perinatal brain injury. **a** Experimental paradigm. BrdU was administered either from P11 to P15 (**c**) or P24−P28 (**d**). **b** Experimental groups**. c** Quantification of total EGFP + BrdU + CC1 + cells at P45, following BrdU injections immediately after HX (P11−P15) (*n* = 3 per group) (from left to right: ***p = 0.0006, ***p = 0.0008). **d** Quantification of total EGFP + BrdU + CC1 + cells at P45, following BrdU injections during EE (P24−P28) (NX, *n* = 5; NX-EE, *n* = 4; HX, *n* = 5; HX-EE, *n* = 4) (****p *<* 0.0001, ***p = 0.0008). Data expressed as mean ± SEM. One-way ANOVA followed by a Tukey’s post hoc test for multiple comparisons was used to compare differences between groups. Source data are provided as a Source Data file.

Recovery from HX in EE led to significant increases in OPCs and myelin proteins, suggesting that EE enhances the generation of new OLs. To evaluate if EE modulates oligodendrogenesis, mice received intraperitoneal injections of BrdU during early periods of recovery to label proliferating OPCs, followed by quantification of BrdU^+^CC1^+^ newly generated OLs at P45. First, we evaluated the fate of proliferating OPCs in subcortical WM immediately following HX. Here, we injected BrdU from P11 to P15, a period succeeding HX, but preceding EE. This led to an increase in EGFP^+^BrdU^+^CC1^+^ cells at P45 in both groups exposed to HX (HX vs. HX-EE vs. NX: 21.1 ± 0.96 cells/mm^3^ vs. 20.81 ± 1.39 vs. 11.59 ± 0.74) (Fig. 2a–c), but no changes induced specifically by EE. Next, BrdU was administered during enrichment (P24−P28), resulting in an increased number of EGFP^+^BrdU^+^CC1^+^cells at P45 in HX-EE mice, compared to HX (23.75 ± 0.55 cells/mm^3^ vs. 20.64 ± 0.45) (Fig. 2d). These results affirm the generation of new OLs during the week following HX—and importantly—additional oligodendrogenesis induced by EE under pathological conditions.

Interestingly, EE did not promote OL generation under normal physiological (NX) conditions. It is possible that since we employed NX-EE after the period of maximal OPC proliferation that occurs during normal development^32^, initiation of EE at P15 had little influence on the normally maturing OPC population. However, since EE is known to stimulate hippocampal neurogenesis^33–35^, we sought to validate our EE protocol under NX conditions. To this end, we quantified neural stem cell and progenitor proliferation in the dentate gyrus (DG) of animals reared in NX or NX-EE (Supplementary Fig. 4a, b). In NX-EE mice, the number of Sox2^+^ and Nestin^+^ stem and progenitor cells were significantly increased compared to NX at P45 (Sox2: 10.6 ± 2.6 %cells/DAPI vs. 4.1 ± 0.7; Nestin: 6.2 ± 1 vs. 2.3 ± 0.2) (Supplementary Fig. 4c, d). This corresponded with an increase in proliferating Ki67^+^ cells in NX-EE mice (2.0 ± 0.3 % cells/DAPI vs. 0.8 ± 0.3) (Supplementary Fig. 4e). Lastly, we observed an increase in Doublecortin (Dcx)^+^ neuronal progenitors in the NX-EE group compared to NX (13.4 ± 1.4 % cells/DAPI vs. 6.5 ± 1.1) (Supplementary Fig. 4f). Therefore, our EE paradigm induces neurogenesis under normal physiological conditions. Since there is a selective effect of EE on OL generation only under HX conditions, we hypothesize that HX opens a critical period in which EE can beneficially influence developmental myelination.

### EE promotes functional recovery from hypoxia

**Fig. 3:**
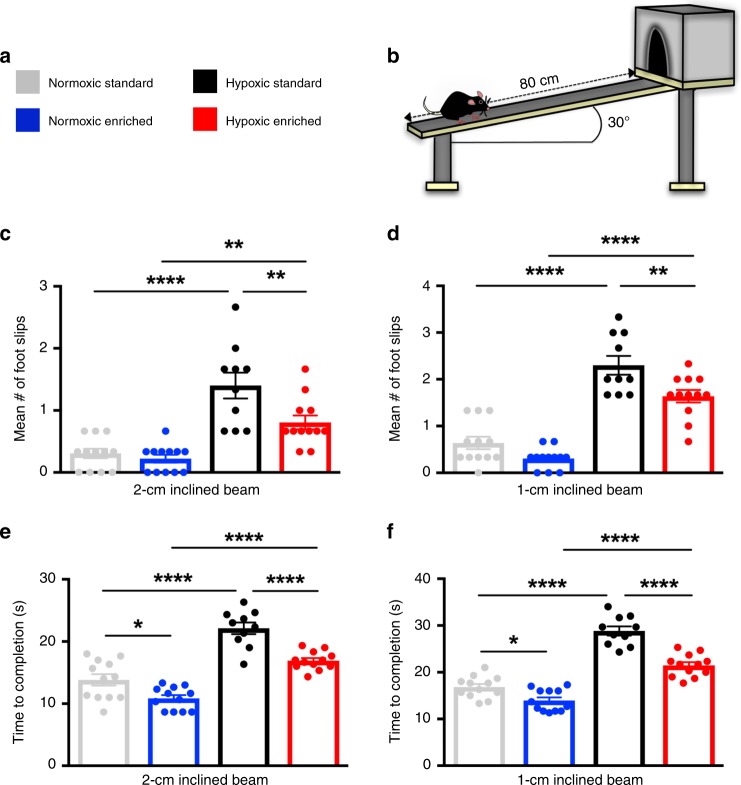
Environmental enrichment promotes functional recovery after perinatal brain injury. **a**, **b** Experimental groups tested on the 2-cm and 1-cm width inclined beam-walking task. **c** Quantification of foot slips for the 2-cm beam at P45 (from left to right: ****p < 0.0001, **p = 0.005, **p = 0.007) (NX, *n* = 12; NX-EE, *n* = 12; HX, *n* = 10; HX-EE, *n* = 12). **d** Quantification of foot slips for the 1-cm inclined beam at P45 (****p < 0.0001, **p = 0.009) (NX, *n* = 12; NX-EE, *n* = 12; HX, *n* = 10; HX-EE, *n* = 12). **e** Quantification of time to completion for the 2-cm beam at P45 (*p = 0.02, ****p < 0.0001) (NX, *n* = 12; NX-EE, *n* = 12; HX, *n* = 10; HX-EE, *n* = 12). **f** Quantification of time to completion for the 1-cm inclined beam at P45 (*p = 0.04, ****p < 0.0001) (NX, *n* = 12; NX-EE, *n* = 12; HX, *n* = 10; HX-EE, *n* = 12). Data expressed as mean ± SEM. One-way ANOVA followed by a Tukey’s post hoc test for multiple comparisons was used to compare differences between groups. Source data are provided as a Source Data file.

Based on cellular findings suggesting a regenerative response after HX and subsequent EE, we investigated EE-induced functional recovery using a subcortical WM-dependent behavioral challenge—the inclined beam-walking task^8,36^ (Fig. 3). In this task, mice traverse a inclined beam while the number of foot slips are indicative of performance. This test is a useful and reproducible assessment of motor coordination and balance, and has been utilized in a number of developmental, injury, and genetic studies^8,37–39^. To vary the level of difficulty, beams of either 2-cm width or 1-cm width were used.

As previously shown^8^, CNP-EGFP mice exposed to HX alone performed poorly on the inclined beam task vs. NX (2-cm: 1.4 ± 0.2 slips vs. 0.3 ± 0.1; 1-cm: 2.3 ± 0.2 vs. 0.6 ± 0.1) (Fig. 3c, d). However, mice exposed to HX and subsequent EE had significantly fewer foot slips on both 2-cm and 1-cm beams at P45 (2-cm: 0.8 ± 0.1 slips vs. 1.4 ± 0.2; 1-cm: 1.6 ± 0.1 vs. 2.3 ± 0.2), indicating improvement in motor coordination and balance. No differences in foot slips were detected at either beam width in NX groups; however, NX-EE mice traversed the beam in a shorter amount of time (Fig. 3e, f). These behavioral studies strongly align with our cellular findings, suggesting that continuous EE ameliorates the effects of perinatal HX by increasing OL maturation and promoting myelination required for WM-dependent functional improvement.

### A complete EE is necessary for recovery from hypoxia

**Fig. 4:**
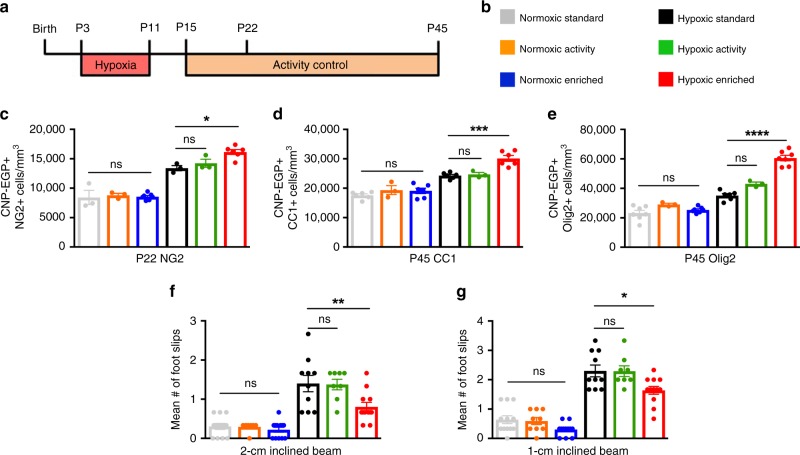
Locomotor activity alone had no effects on OL maturation and did not improve functional recovery after perinatal brain injury. **a** Experimental paradigm. Activity control consisted of access to a running wheel. **b** Experimental groups. **c** Quantification of total EGFP + NG2 + OPCs at P22 (*p = 0.015) (NX, *n* = 3; NX Activity (NX-A), *n* = 3; NX-EE, *n* = 7; HX, *n* = 3; HX Activity (HX-A), *n* = 3; HX-EE, *n* = 6). **d** Quantification of total EGFP + CC1 + OLs at P45 (***p = 0.0002) (NX, *n* = 6; NX-A, *n* = 3; NX-EE, *n* = 6; HX, *n* = 6; HX-A, *n* = 3; HX-EE, *n* = 6). **e** Quantification of total EGFP + Olig2 + cells at P45 (****p < 0.0001)(NX, *n* = 7; NX-A, *n* = 3; NX-EE, *n* = 7; HX, *n* = 6; HX-A, *n* = 3; HX-EE, *n* = 7). **f**, **g** Quantification of foot slips on 2-cm inclined beam (**p = 0.006) (**f**) and 1-cm inclined beam (*p = 0.015) (**g**) at P45 (NX, *n* = 12; NX-A, *n* = 9; NX-EE, *n* = 12; HX, *n* = 10; HX-A, *n* = 8; HX-EE, *n* = 12). Data are expressed as mean ± SEM. One-way ANOVA followed by a Tukey’s post hoc test for multiple comparisons was used to compare differences between groups. Source data are provided as a Source Data file.

To parse out the relative contributions of individual EE components, we first determined the effect of locomotor activity alone during recovery from HX insult (Fig. 4a, b). Aerobic exercise in the form of running influences brain function and increases neurogenesis^40,41^. Further, exercise increases proliferation of NG2^+^ glia in the prefrontal cortex of juvenile mice^42^. For our locomotor activity control, mice were exposed to HX from P3 to P11, as before. However, mice were reared in a cage containing a running wheel, with fewer cagemates (3–5 mice), and without novel objects. We found that access to a running wheel after HX caused no change in OPCs at P22, as well as no change in OLs, OL lineage cells, or myelination at P45 (Fig. 4c–e). Further, running rendered no behavioral improvements in motor coordination and balance after HX brain injury (Fig. 4f, g). These data indicate that voluntary locomotor activity alone is incapable of attenuating the effects of perinatal WM injury.

Studies of premature infants demonstrate that an enhanced social environment positively impacts plasticity and recovery after perinatal brain injury. Preterm infants raised in a two-parent household, particularly one in which the level of maternal education is high, show improved neurodevelopmental outcomes^9^. Conversely, preterm infants reared in private rooms show delayed neurological maturation vs. infants cared for in larger wards^43^. Also, social isolation in juvenile mice causes abnormally thin myelin sheaths and reduced myelin sheath number in the prefrontal cortex, resulting in working memory and sociability deficits^44^. This suggests that myelination can be manipulated by the social environment, and that social dynamics play an important role in the neurological outcome of children born preterm.

To evaluate the effects of socialization and novel stimuli, mice were separated into experimental groups based on exposure to either NX or HX, as well as increased socialization (8−12 mice per cage) and novel object exposure (no access to a running wheel) (Supplementary Fig. 5a, b). Interestingly, these mice had no differences in proliferating OPC, OLs, or total OL lineage cells compared to HX controls at P45 (Supplementary Fig. 5c–e). Therefore, a complete enriched environment (locomotor activity, increased socialization, and novel object exposure) is required to promote recovery from perinatal brain injury.

### A critical neurodevelopmental window for EE after hypoxia

**Fig. 5:**
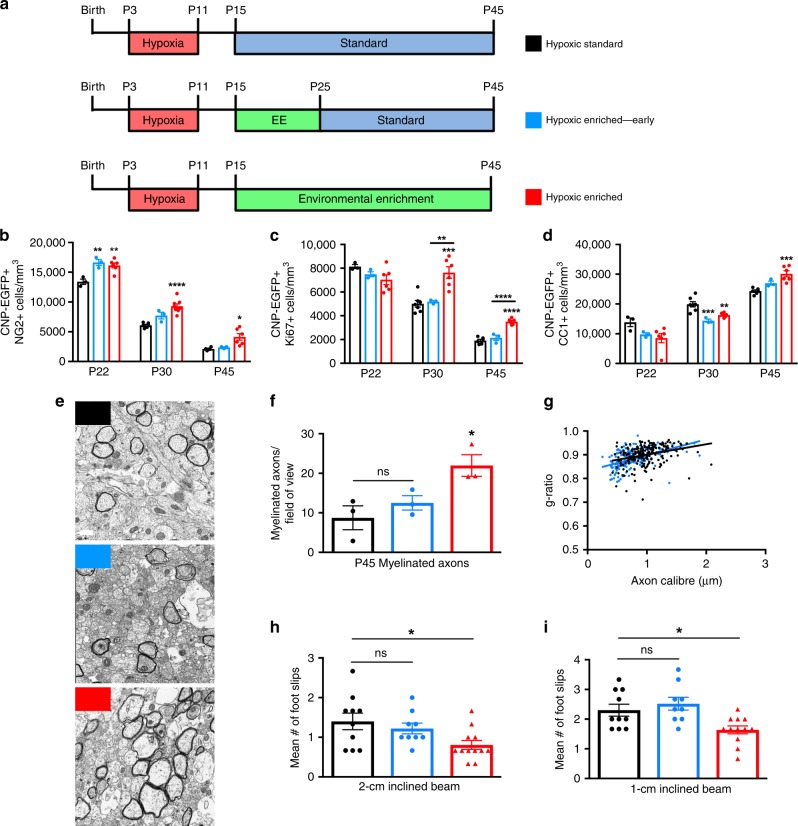
Continuous environmental enrichment is required for functional white matter recovery after perinatal brain injury. **a** Experimental paradigms. **b** Quantification of EGFP + NG2 + OPCs (P22: HX, *n* = 3; HX-EE early, *n* = 3; HX-EE continuous, *n* = 6; P30: HX, *n* = 6; HX-EE early, *n* = 3; HX-EE continuous, *n* = 8; P45: HX, *n* = 4; HX-EE early, *n* = 3; HX-EE continuous, *n* = 6) (from left to right: **p = 0.006, **p = 0.007, ****p *<* 0.0001, *p *=* 0.023). **c** Quantification of EGFP + Ki67 + OPCs (at P22: HX, *n* = 3; HX-EE early, *n* = 3; HX-EE continuous, *n* = 6; at P30: HX, *n* = 7; HX-EE early, *n* = 3; HX-EE continuous, *n* = 6; at P45: HX, *n* = 6; HX-EE early, *n* = 3; HX-EE continuous, *n* = 6) (**p = 0.005, ***p *=* 0.0004, ****p *<* 0.0001). **d** Quantification of EGFP + CC1 + OLs (at P22: HX, *n* = 3; HX-EE early, *n* = 3; HX-EE continuous, *n* = 6; at P30: HX, *n* = 7; HX-EE early, *n* = 3; HX-EE continuous, *n* = 6; at P45: HX, *n* = 6; HX-EE early, *n* = 3; HX-EE continuous, *n* = 6) (from left to right: ***p = 0.001, **p = 0.005, ***p = 0.0003). **e** Representative EM images from P45 white matter. Scale bar = 0.5 μm. **f** Quantification of myelinated axon number (*n* = 3 animals per group) (*p = 0.025; ns = not significant). **g** Scatter plot of g-ratios in HX vs. HX-Continuous EE, and HX vs. HX-Early EE (*n* = 3 animals per group). **h**, **i** Quantification of foot slips on 2-cm inclined beam (**h**) and 1-cm inclined beam (**i**) at P45 (HX, *n* = 10; HX-EE early, *n* = 9; HX-EE continuous, *n* = 12) (2-cm: *p = 0.025, 1-cm: *p *=* 0.032). Data are expressed as mean ± SEM. One-way ANOVA followed by a Tukey’s post hoc test for multiple comparisons was used to compare differences between groups at each age. Source data are provided as a Source Data file.

The existence of critical windows for cellular and circuit plasticity during development highlights a need to refine the timing of EE intervention^45^. To determine the optimal timing and length of intervention, mice recovered in EE during different neurodevelopmental periods. In the early intervention paradigm (Fig. 5a), mice were exposed to EE from P15 to P25. In the delayed intervention paradigm, mice were reared in EE from P25 to P35 (Supplementary Fig. 6e). For the late intervention paradigm, mice were housed in EE from P35 to P45 (Supplementary Fig. 6i). Finally, in the delayed and extended group, mice were initially housed in a standard environment until a 30-day exposure to EE from P25 to P55, mimicking the length of the original continuous paradigm (Supplementary Fig. 6m).

Interestingly, with early intervention only, we found an increase in the number of OLs (26,997 ± 690 cells/mm^3^ vs. 24,302 ± 411; Supplementary Fig. 6c) and OL lineage cells (49,005 ± 1499 cells/mm^3^ vs. 36,448 ± 1573; Supplementary Fig. 6d) at P45. However, OLs were not increased to the same extent as they were after continuous EE (30,115 ± 1011 cells/mm^3^ vs. 26,997 ± 690; Fig. 5d). Additionally, the cellular changes induced by early intervention did not confer any ultrastructural or functional improvements (Fig. 5e–i; Supplementary Fig. 7a–c). Unlike the continuous and early EE paradigms, delayed and late interventions yielded no significant cellular changes in OL lineage cells compared to HX controls (Supplementary Fig. 6e–l). These mice also displayed no behavioral improvements on the inclined beam-walking task (Supplementary Fig. 7d–i). Importantly, gender differences were not evident in behavioral performance in our alternative neurodevelopmental paradigms of EE (Supplementary Fig. 8).

**Fig. 6:**
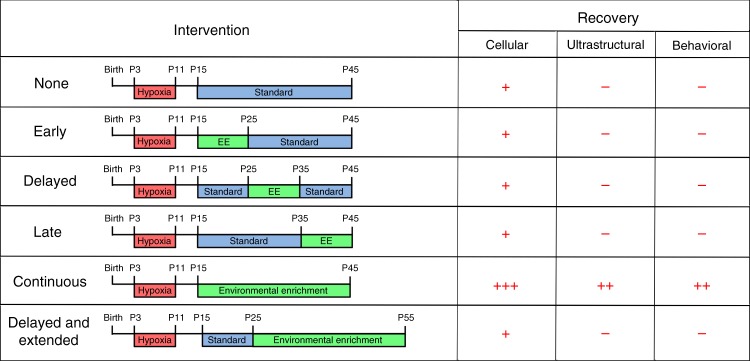
Summary of cellular, ultrastructural, and behavioral recovery after perinatal brain injury using distinct neurodevelopmental paradigms of environmental enrichment. Alternative paradigms evaluated exposure to HX and subsequent EE after no intervention, early intervention (P15−P25), delayed intervention (P25−P35), late intervention (P35−P45), or delayed and extended intervention (P25−P55), with mice otherwise reared in standard laboratory housing.

Lastly, to determine if the length of EE was the primary factor driving EE-induced recovery from HX, mice were given 30 days of EE—the same as the continuous paradigm—but exposure was delayed 10 days (Supplementary Fig. 6m). Again, this intervention paradigm caused no significant cellular changes in OL lineage cells and no behavioral improvements between HX and HX-EE groups (Supplementary Figs. 6n–p and  7k, l). Interestingly, the delayed and extended EE paradigm caused a significant increase in proliferating OPCs in NX-EE mice. This increase in OPC proliferation, which is not present in the continuous EE paradigm, suggests that EE-induced effects on OPC proliferation in normally developing mice is age dependent. Still, this paradigm of EE is unlikely to have caused any changes in subcortical WM myelination, because behavioral performance on the inclined beam was unchanged (Supplementary Fig. 7j–l). The cellular, ultrastructural, and behavioral results obtained with distinct paradigms of EE during different developmental periods after HX are summarized in Fig. 6.

### De novo OL generation is required for EE-induced recovery

**Fig. 7:**
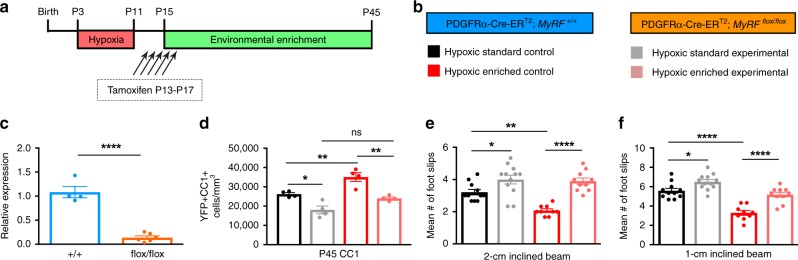
De novo oligodendrocyte generation is required to promote functional recovery induced by environmental enrichment. **a** Experimental paradigm. Mice received tamoxifen once daily from P13 to P17. **b** Experimental groups. **c** Quantitative PCR for *MyRF* mRNA levels in white matter of wild-type (MyRF^+/+^, *n* = 4) and knockdown (MyRF^fl/fl^, *n* = 6) mice at P30 (two-tailed unpaired *t* test, ****p < 0.0001). **d** Quantification of total YFP + CC1 + OLs at P45 (*n* = 4 per group) (from left to right: *p = 0.015, **p *=* 0.009, **p = 0.002). **e**, **f** Quantification of foot slips on 2-cm inclined beam (*p *=* 0.04, **p *=* 0.003, ****p < 0.0001) (**e**) and 1-cm inclined beam (*p *=* 0.04, ****p < 0.0001) (**f**) at P45 (MyRF^+/+^ HX Control, *n* = 11 animals; MyRF^+/+^ HX-EE Co*n*trol, *n* = 9 animals; MyRF^fl/fl^ HX Experimental, *n* = 12 animals; MyRF^fl/fl^ HX-EE Experimental, *n* = 10 animals). Data are expressed as mean ± SEM. One-way ANOVA followed by a Tukey’s post hoc test for multiple comparisons was used to compare differences between groups. Source data are provided as a Source Data file.

To confirm that WM-dependent behavioral improvements following EE require de novo oligodendrogenesis, we conditionally ablated expression of myelin regulatory factor (MyRF) in OPCs, effectively blocking OL maturation^46,47^. PDGFRα-CreER^T2^ transgenic mice on a ROSA26-YFP background were crossed with MyRF^(flox/flox)^ mice to obtain PDGFRα-CreER^T2^: R26R-YFP: MyRF^(flox/flox)^ mice and PDGFRα-CreER^T2^: R26R-YFP:MyRF^(+/+)^ controls (Fig. 7a, b). Recombination at the MyRF locus was confirmed by qPCR (*p* < 0.0001, unpaired *t* test; Fig. 7c). Focusing on the role of EE after injury, transgenic mice were separated into two experimental groups for both controls (MyRF^(+/+)^) and mutants (MyRF^(fl/fl)^)—(1) HX, and (2) HX-EE. These animals were exposed to HX from P3 to P11, and subsequently reared in continuous EE from P15 to P45. In HX-EE MyRF^(+/+)^ controls, EE significantly increased YFP^+^CC1^+^ OLs compared to MyRF^(+/+)^ controls undergoing HX alone (35,051 ± 2255 cells/mm^3^ vs. 26,209 ± 750) (Fig. 7d). These data are consistent with that found using the continuous EE paradigm in CNP-EGFP mice. However, HX-EE MyRF^(fl/fl)^ mutants had a significantly reduced number of YFP^+^CC1^+^ OLs, compared to HX-EE MyRF^(+/+)^ controls (24,032 ± 678 cells/mm^3^ vs. 35,051 ± 2255) (Fig. 7d). This indicates that the increase in OLs normally seen after EE requires de novo oligodendrogenesis. We next assessed motor coordination in MyRF^(+/+)^ controls and MyRF^(fl/fl)^ mutants after HX using the inclined beam-walking test. Importantly, HX-EE MyRF^(+/+)^ mice outperformed their HX-standard-housing counterparts at P45 on the 2-cm and 1-cm inclined beam task (2-cm: 2.1 ± 0.1 slips vs. 3.2 ± 0.2; 1-cm: 3.3 ± 0.3 vs. 5.6 ± 0.3), and MyRF inactivation negated this WM-dependent behavioral improvement (2-cm: 3.9 ± 0.2 slips vs. 2.1 ± 0.1; 1-cm: 5.2 ± 0.3 vs. 3.3 ± 0.3) (Fig. 7e, f). The number of foot slips performed by HX-EE MyRF^(fl/fl)^ mice was not significantly different than HX MyRF^(+/+)^ mice (2-cm: 3.9 ± 0.2 slips vs. 3.2 ± 0.2; 1-cm: 5.2 ± 0.3 vs. 5.6 ± 0.3), indicating that blocking de novo oligodendrogenesis completely prevents EE-induced functional recovery from perinatal brain injury.

### EE alters OPC and OL molecular properties after hypoxia

**Fig. 8:**
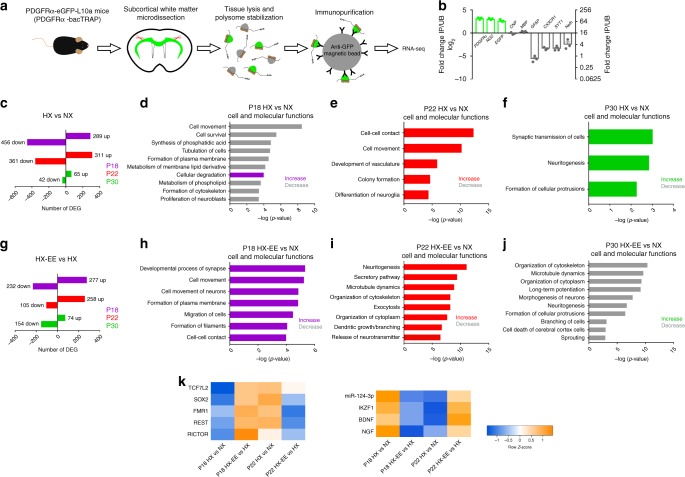
RNA-seq reveals EE-induced changes in OPC translatome following perinatal hypoxia. **a** Schematic representation of experimental design and TRAP methodology. **b** qRT-PCR analysis of immunoprecipitated (IP) and unbound (UB) fractions from the white matter of PDGFRɑ-bacTRAP mice. Green bars correspond to genes enriched in OPCs, and grayscale bars correspond to genes enriched in other cell types. Bars represent mean ± SEM (*n* = 3 per group). Platelet-derived growth factor receptor alpha (*Pdgfrα*), neural/glial antigen 2 (*NG2*), enhanced green fluorescent protein (*Egfp*), *Cnp*, myelin basic protein (*Mbp*), glial fibrillary acidic protein (*Gfap*; astrocytes), fractalkine receptor (*Cx3cr1*; microglia), synaptotagmin-1 (*Syt1*; neurons), and neurofilament-heavy (*Nefh*; axons). **c** Number of DEGs between HX and NX mice at P18, P22, and P30 (Wald test with Benjamini−Hochberg post hoc, adjusted *p* < 0.05, normalized counts > 5) (P18: HX, *n* = 4, NX, *n* = 3; P22: HX, *n* = 5, NX, *n* = 6; P30: HX, *n* = 3, NX, *n* = 6). **d**–**f** Predicted increases (colored) and decreases (gray) in cell and molecular functions (determined by directional *z*-scores) in HX compared to NX at P18 (**d**), P22 (**e**), and P30 (**f**). Cell and molecular functions ranked based on *p* value as determined using Fisher’s Exact Test. **g** Number of DEGs between HX-EE and HX mice at P18, P22, and P30 (Wald test with Benjamini−Hochberg post hoc, adjusted *p* < 0.05, normalized counts > 5) (P18: *n* = 4 per group; P22: *n* = 5 per group; P30: HX-EE, *n* = 5, HX, *n* = 3). **h**–**j** Predicted increases (colored) and decreases (gray) in cell and molecular functions (determined by directional *z*-scores) in HX-EE compared to HX at P18 (**h**), P22 (**i**), and P30 (**j**). Cell and molecular functions ranked based on *p* value as determined using Fisher’s Exact Test. **k** Heatmaps of most significantly predicted upstream regulators across four comparisons (P18 HX vs. NX, P18 HX-EE vs. HX, P22 HX vs. NX, and P22 HX-EE vs. HX). Boxes are colorized with *z*-scores (orange = activated, blue = inactivated). Source data are provided as a Source Data file.

A key question remaining is what causes the selective effect of EE-induced recovery in the OL lineage? To begin to address this question, we utilized TRAP and RNA-seq to isolate translating mRNAs specifically from WM OPCs or OLs during normal development (NX), after HX, or after HX-EE. This strategy employs a transgenic mouse line engineered to selectively tag the L10a ribosomal subunit of OPCs or OLs with green fluorescent protein (GFP)^48^. We used transgenic lines under the control of the mouse platelet-derived growth factor receptor alpha promotor (PDGFRɑ-bacTRAP), or CNP promoter (CNP-bacTRAP). This allowed for isolation of actively translating, polyribosome-associated mRNAs from OPCs, or differentiated OLs in an in vivo cellular environment at crucial stages of recovery (Fig. 8a).

**Fig. 9:**
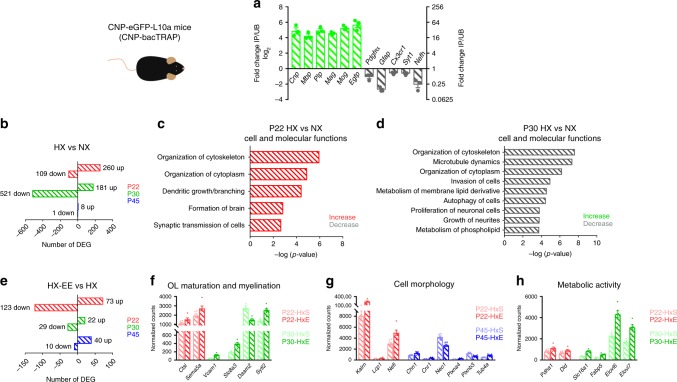
RNA-seq reveals EE-induced changes in oligodendrocyte translatome following perinatal hypoxia. **a** qRT-PCR analysis of immunoprecipitated (IP) and unbound (UB) fractions from the white matter of CNP-bacTRAP mice. Green bars correspond to genes enriched in OLs, and grayscale bars correspond to genes enriched in other cell types. Bars represent mean ± SEM (*n* = 3 per group). *Cnp*, myelin basic protein (*Mbp*), proteolipid protein (Plp), myelin-associated glycoprotein (*Mag*), myelin oligodendrocyte glycoprotein (*Mog*), enhanced green fluorescent protein (*Egfp*), platelet-derived growth factor receptor alpha (*Pdgfrα*), glial fibrillary acidic protein (*Gfap*; astrocytes), fractalkine receptor (*Cx3cr1*; microglia), synaptotagmin-1 (*Syt1*; neurons), and neurofilament-heavy (*Nefh*; axons). **b** Number of DEGs between HX and NX mice at P22, P30 and P45 (Wald test with Benjamini−Hochberg post hoc, adjusted *p* < 0.05, normalized counts > 5) (P22: HX, *n* = 6, NX, *n* = 4; P30: HX, *n* = 3, NX, *n* = 5; P45: HX, *n* = 6, NX, *n* = 5). **c**, **d** Predicted increases (colored) and decreases (gray) in cell and molecular functions (determined by directional *z*-scores) in HX compared to NX at P22 (**c**), and P30 (**d**). Cell and molecular functions ranked based on *p* value as determined using Fisher’s Exact Test. **e** Number of DEGs between HX-EE and HX mice at P22, P30 and P45 (Wald test with Benjamini−Hochberg post hoc, adjusted *p* *<* 0.05, normalized counts > 5) (P22: *n* = 6 per group; P30: HX-EE, *n* = 6, HX, *n* = 3; P45: *n* = 6 per group). **f**–**h** Normalized counts of a selection of significant genes (Wald test with Benjamini−Hochberg post hoc, adjusted *p* < 0.05) involved in OL maturation and myelination (**f)**, cell morphology (**g**), and metabolic activity (**h**) compared between HX-EE and HX at P22 (red), P30 (green), and P45 (blue) (P22: *n* = 6 per group; P30: HX-EE, *n* = 6, HX, *n* = 3; P45: *n* = 6 per group). Bars represent mean ± SEM. Source data are provided as a Source Data file.

Confocal imaging of the subcortical WM from PDGFRɑ-bacTRAP and CNP-bacTRAP mice confirmed that all GFP-expressing cells coexpressed the OL lineage marker OLIG2 (Supplementary Fig. 10a, c). In the PDGFRɑ-bacTRAP mice, ~72% of GFP^+^ cells coexpressed NG2, ~57% of GFP^+^ cells coexpressed PDGFRɑ, and ~48% of GFP^+^ cells coexpressed CC1, indicating that captured mRNAs are from OPCs as well as immature OLs (Supplementary Fig. 10a, b). In the CNP-bacTRAP mice, GFP expression was restricted to CC1-expressing OLs, and not OPCs (Supplementary Fig. 10c, d). To demonstrate that the TRAP technique isolated OPC- and OL-specific mRNA, we performed qRT-PCR comparing immunoprecipitated (IP) and unbound fractions from the WM (Figs. 8b and 9a). This analysis revealed that the IP fraction from PDGFRɑ-bacTRAP mice was enriched for OPC-specific mRNAs, whereas the IP fraction from CNP-bacTRAP mice was enriched for myelin-specific mRNAs, as well as EGFP. Unbound fractions from both mouse lines were enriched for mRNA of other cell types such as astrocytes, microglia, neurons, and axons.

Next, we replicated major findings from the CNP-EGFP mice in the bacTRAP mice using the complete and continuous EE paradigm. First, we assessed the effects of HX on OPCs and proliferating OPCs vs. NX controls in CNP-bacTRAP mice. As previously shown, there was an increase in both cell types at P30 in response to injury (NG2^+^: 14,325 ± 760 cells/mm^3^ vs. 7813 ± 1036; NG2^+^Ki67^+^: 3781 ± 351 vs. 2263 ± 443; Supplementary Fig. 11c, d). Furthermore, EE enhanced this endogenous response and significantly increased OPCs and proliferating OPCs after HX exposure (NG2^+^: 18,759 ± 1595 cells/mm^3^ vs. 14,325 ± 760; NG2^+^Ki67^+^: 5273 ± 760 vs. 3781 ± 351), but had no effect on NX mice (NX and NX-EE). We next investigated corresponding changes in OLs at P45, and found a significant increase in the total number of CC1^+^ OLs in our HX-EE group compared to HX (83,822 ± 5633 cells/mm^3^ vs. 62,941 ± 5362; Supplementary Fig. 11e), confirming a profound OL response to EE following HX. Using the inclined beam-walking task, we also confirmed that EE promoted functional recovery after perinatal HX in both PDGFRɑ- and CNP-bacTRAP mice. HX mice performed poorly on the inclined beam task vs. NX controls. But mice exposed to HX and subsequent EE exhibited fewer foot slips on both the 2-cm and 1-cm beam at P45 (Supplementary Fig. 11f–i), indicating significant improvements in motor coordination and balance. No significant changes in the number of foot slips were detected between NX groups. Altogether, these results aligned with our findings in CNP-EGFP mice, and confirm the cellular and behavioral effects of HX and subsequent EE in the bacTRAP mice.

Using PDGFRɑ-bacTRAP mice and RNA-seq, we first determined how HX impacted normal OPC molecular properties at various time points after HX (Fig. 8a, c–f). Differential gene expression analysis between HX and NX OPCs revealed 745 differentially expressed genes (DEGs) at P18 (289 upregulated, 456 downregulated), 672 DEGs at P22 (311 up, 361 down), and 107 DEGs at P45 (65 up, 42 down; Fig. 8c and Supplementary Data 1–3). All reported DEGs have normalized read counts above 5 in all samples, and reach statistical significance (adjusted *p* *<* 0.05; Wald test with Benjamini−Hochberg post hoc). Next, we used Ingenuity Pathway Analysis (IPA; Qiagen) to identify biological processes implicated by the vast changes in the OPC translatome after HX. At P18, analyses predicted significant changes in numerous cell and molecular processes indicative of abnormal OPC functions. These include decreased cell movement, cell survival, plasma membrane formation, and cytoskeletal formation (Fig. 8d). However, at P22 and P30, there are predicted increases in processes that, in the context of OPC development and maturation, can be interpreted as axon-OPC synapse formation, OPC differentiation, and initial myelin formation and extension. These include cell movement, cell−cell contact, differentiation of neuroglia, and formation of cellular protrusions (Fig. 8e–f). This reversal of predicted functions suggests that OPCs rebound from an initial HX insult and upregulate maturation programs that are no longer as active in normally developing OPCs.

To elucidate OPC-specific mechanisms involved in the EE-induced recovery from perinatal hypoxia, we next compared the translatomes of HX-EE and HX OPCs. Differential gene expression analysis uncovered substantial EE-induced changes that yielded 509 DEGs at P18 (277 up, 232 down), 363 DEGs at P22 (258 up, 105 down), and 228 DEGs at P30 (74 up, 154 down; Fig. 8g and Supplementary Data 4–6). Interestingly, IPA revealed that EE induced a temporal shift in cell and molecular properties of OPCs. Whereas HX OPCs decreased various potential functions at P18 that were reversed at P22 and P30, HX-EE OPCs at P18 and P22 increased many of the same functions (i.e. synapse formation, membrane formation, and process extension; Fig. 8h–i). Furthermore, these functions were decreased in HX-EE OPCs at P30 (Fig. 8j), suggesting that EE causes a transient increase in OPC-maturation processes that are impaired or delayed in HX. To provide additional evidence of transient changes in potential OPC functions, we compared predicted upstream regulators of our DEGs across NX, HX, and HX-EE OPCs (Fig. 8k). As expected, a pattern of temporary upstream regulator activation (orange) or inhibition (blue) emerged. Most of these upstream regulators have known effects on OL development, highlighting the complex and multifaceted molecular mechanisms at play in OPCs recovering from HX. Together, these data indicate that recovery from HX in EE accelerates the endogenous molecular response and reverses impaired biological processes of OPCs.

Nevertheless, the sequencing data from PDGFRɑ-bacTRAP mice comes short of explaining the HX-induced deficits in myelination that are rescued by EE. Thus, we utilized CNP-bacTRAP mice to uncover mechanisms of EE-induced recovery from HX that are specific to a more mature population of OLs. As before, we performed differential gene expression analysis between HX and NX OLs, to determine how HX impacted normal OL functions at various time points after HX. At P22, there were 369 DEGs (260 up, 109 down), 702 DEGs at P30 (181 up, 521 down), and 9 DEGs at P45 (8 up, 1 down; Fig. 9b and Supplementary Data 7–9). The fact that differences in the OL translatome are virtually eliminated by P45 despite persistent myelin deficits provides additional evidence of a critical period of intervention. Based on IPA, HX OLs at P22 had increased cell and molecular functions such as cytoskeletal organization, dendritic growth, and synaptic transmission compared to NX (Fig. 9c). These results likely reflect a less mature OL population that, similar to the OPC population, is undergoing delayed maturation. However, at P30, HX OLs had decreased organization of cytoskeleton, microtubule dynamics, neurite growth, and phospholipid metabolism (Fig. 9d). In the context of differentiated OLs, these data indicate a severe impairment in the ability of OLs to form myelin and wrap axons after HX. Furthermore, these data align with our previous findings that HX causes an accumulation of premyelinating OLs (Fig. 1m, n).

To uncover EE-induced mechanisms responsible for the rescue of myelination following HX, we next compared the translatomes of HX-EE and HX OLs. Differential gene expression analysis revealed 196 DEGs at P22 (73 up, 123 down), 51 DEGs at P30 (22 up, 29 down), and 50 DEGs at P45 (40 up, 10 down; Fig. 9e and Supplementary Data 10–12). Because this comparison yielded fewer DEGs than in all previous data sets, IPA yielded minimal results. Nevertheless, we manually identified numerous DEGs with similar biological functions (Fig. 9f, g). The first group of DEGs identified in HX-EE OLs were genes implicated in OL maturation or myelination at P22 and P30 (Fig. 9f). Genes such as cathepsin L (*Ctsl*), semaphorin 5A (*Sema5a*), and synaptotagmin-like 2 (*Sytl2*) are highly expressed by differentiating OLs^49^, whereas vascular cell-adhesion molecule (*Vcam1*), sodium-calcium exchanger-solute carrier family 8 member A3 (*Slc8a3*), and disheveled-associated activator of morphogenesis 2 (*Daam2*) play a causal role in OL differentiation or myelination^50–52^.

Next, we identified multiple genes involved in cellular morphology that were differentially expressed at P22 and P45 (Fig. 9g). At P22, kalirin RhoGEF kinase (*Kalrn*), plastin 2 (*Lcp1*), and neurofilament light (*Nefl*) were all upregulated in HX-EE OLs. At P45, HX-EE induced differential expression of chimerin 1 (*Chn1*), cannabinoid receptor 1 (*Cnr1*), and plexin-A4 (*Plxna4*), neogenin 1 (*Neo1*), and plexin-B3 (*Plxnb3*), and tubulin alpha 4a (*Tuba4a*). All of the proteins that these DEGs express are involved in cytoskeletal dynamics and cell protrusions. While most of the evidence supporting the involvement of these proteins in process outgrowth lies in neurons, cytoskeletal dynamics are essential for OL health, survival, and myelination, and therefore likely play a key role in neurological recovery^53–56^.

Lastly, a group of upregulated genes involved in metabolic activity were identified in HX-EE OLs (Fig. 9h). At P22, pyruvate dehydrogenase E1 alpha 1 subunit (*Pdha1*) and dihydrolipoamide dehydrogenase (*Dld*) were both increased in HX-EE OLs. These enzymes are required for the pyruvate dehydrogenase complex (PDC), which produces acetyl-CoA and is therefore essential to cellular energetics. At P30, the lactate transporter, *Slc16a1*, also known as monocarboxylate transporter 1 (MCT1), was upregulated in HX-EE OLs. This vital transporter is known to provide dynamic metabolic support to axons from OLs^57,58^. Therefore, HX-EE OLs have an increased capacity to support intrahemispheric axons traversing the subcortical white matter, which play a key role in cognition and behavior during childhood and adolescence^59^. Finally, fatty acid elongases 6 and 7 (*Elovl6* and *Elovl7*), and fatty acid binding protein 5 (*Fabp5*)—three genes involved in lipid metabolism^60^—are upregulated at P30. Due to the high demand for lipids during myelination, and because metabolism of membrane lipids is one of the top processes that is decreased in OLs after HX, it is likely that these genes are important drivers of myelin production after injury.

Altogether, our OPC- and OL-specific RNA-sequencing results provide strong mechanistic evidence of the dynamic molecular changes underlying cellular, anatomical, and functional recovery from perinatal HX induced by EE. Our findings also provide a wealth of new information regarding complex changes in specific gene networks that govern OL function after HX. Importantly, we offer many exciting avenues of scientific exploration to further decipher the beneficial impact of EE on perinatal brain injury at the cellular and molecular levels.

## Discussion

Premature birth is a significant public health concern because of the increasingly large number of infants that survive with permanent neurodevelopmental disability and behavioral abnormalities^1,2,61^. However, these neurological morbidities can be substantially improved by social, activity, and environmental influences^25,47,62^.

EE—locomotor activity, increased socialization, and novel object exposure—attenuates the effects of perinatal brain injury and promotes OL regeneration. This effect on OL maturation ultimately results in EE-induced enhanced myelination, leading to WM-specific behavioral recovery after HX injury. In line with previous findings^44^, our EE protocol did not promote oligodendrogenesis and myelination under normal physiological conditions. It is likely then that perinatal injury selectively opens a developmental window of myelin plasticity, during which environmental influence enhances endogenous repair and restores behavioral function. To this end, EE can be used as a modulator of neurorehabilitation in premature brain injury.

While the developmental trajectory of preterm infants remains complicated, timing of intervention after insult is certainly a crucial factor. Imaging studies have substantiated that the extent of chronic cognitive and behavioral deficits is correlated to the gestational age of the infant and the degree of WM injury^63,64^. Unfortunately, the pathogenesis of WM injury typically coincides with a critical interval of gestational immaturity^3^ and a prolific subset of HX-sensitive OPCs diffusely populating the brain^65^. However, this period is also marked by a heightened sensitivity to both the structural remodeling of myelin, and a functional adaptation of OLs in response to injury^4^. By investigating distinct time-sensitive paradigms of EE, we identified critical periods of OL plasticity-mediated recovery, and therefore established the optimal timing and duration of EE-based intervention. Our data is in line with a known epoch of OL recovery and plasticity seen after HX brain injury in rodents^8^ that coincides with reconstitution of cortical neurogenesis after HX^66^. Additionally, studies have demonstrated a strong proliferative response following HX, suggesting OL regeneration after insult^17^. Accordingly, EE seems to amplify this endogenous reparative response. Importantly, only early and continuous EE ameliorated the cellular, ultrastructural, and behavioral deficits incurred from HX, which has significant clinical implications—the sooner EE intervention is given after HX brain injury, the better the outcome. Further, starting intervention too late is substantially ineffective^67^. In this sense, there is a limited temporal window to employ EE after HX. Children lacking positive environmental input in early life are more prone to abnormal neurodevelopment^68^. Early manipulation of the neonatal environment in children born preterm should support and enhance adaptive functional reorganization during a known period of plasticity. Further, evidence suggests that continued implementation of these environmental manipulations in conjunction with continued follow-up care^69^ has lasting effects on functional neurological recovery^11^. Future studies aimed at dissecting the temporal restrictions of an enrichment protocol must strongly consider both timely implementation as well as duration of exposure, both of which can drastically alter neurodevelopmental outcome.

Previous studies show that inactivating MyRF in OPCs blocks maturation, without affecting preexisting OLs or myelin. This results in a near complete failure of OPCs to differentiate into myelinating OLs^70^ and begets OPCs prone to apoptosis^71^, especially after injury^72^. Importantly, this also prevents mice from mastering an activity-dependent task^47^. We utilized this model and found that EE-induced oligodendrogenesis is required for WM-dependent behavioral improvements after HX. Since MyRF is crucial for OPC maturation, its ablation resulted in a decrease in new OLs, even after continuous EE. Further, this negated WM-dependent behavioral improvement. However, our findings in control mice paralleled those seen using continuous EE after HX in CNP-EGFP mice. We therefore present evidence that de novo oligodendrogenesis is required for WM-dependent behavioral recovery.

How might de novo oligodendrogenesis improve behavioral performance? It likely involves multiple pathways and gene networks that promote OPC differentiation and converge upon functional myelination. Our results shed light on the multifaceted cascade of cellular and molecular mechanisms underlying WM injury and identify key enrichment-induced changes in some OPC properties, and in OL maturation and myelination. As such, it is becoming increasingly clear that OLs provide dynamic substrate for the continuous adaptation and refinement of CNS circuitry that occurs in response to diverse physiological demands and environmental influence. Newly generated myelin optimizes circuits that are engaged during sensorimotor tasks, thereby establishing new neuronal connections, strengthening existing ones, and improving circuit integrity in response to experiential input^24,73^. In turn, this would ultimately enhance and restore performance of neural networks after injury to improve behavioral output. The complex interplay between OLs and neurons is not only crucial for normal brain development, but often leads to neurological impairment when compromised. In the absence of functional myelination programs, nerve conduction is impaired, and a variety of neurological disorders ensue^58,74^. MyRF, then, is essential for developmental myelination^46^. This suggests a direct and active requirement for newly generated OLs in EE-induced recovery from HX, and further highlights the importance of MyRF under pathological conditions.

After HX, some newly generated OLs repair myelin in the diffusely injured WM. In doing so, they help to reestablish developmental integrity of the underlying neural circuitry by providing metabolic and physical support to maintain axonal integrity^57,58^. Here, we coupled RNA-sequencing with TRAP to better understand how HX alters normally developing OL lineage cells, and to elucidate mechanisms of EE-induced recovery. This type of in-depth translatomic analysis provides additional insight into the molecular properties of OL lineage cells throughout the time course of injury and recovery. Our results reveal, for the first time, distinct and temporally specific OPC processes that occur after HX. With EE intervention, this endogenous oligodendrogenic response appears to be accelerated in order to resynchronize to normal development. However, EE also induces other processes that are not part of the endogenous repair program. These processes appear to be tightly regulated by a network of regulators known to influence OPC development. Our results highlight the plasticity of OPCs during a critical window following perinatal HX.

Nevertheless, an accelerated timeline of OPC development is insufficient to explain the accumulation of premyelinating OLs and persistent myelin deficits following HX^8^. In fact, data from OL-specific mRNAs indicate that transient impairments of OL cell and molecular functions are sufficient to impair functional recovery and myelination despite normal numbers of OLs. This is an important finding that provides a molecular basis for the critical nature of early intervention. Following recovery from HX in EE, we identified DEGs involved in metabolic and morphological activity. Both of these avenues would surely help to facilitate improved circuit preservation and performance. Since OLs interact with axons and neurons, these metabolic and morphological changes likely alter performance of the neural network to improve behavioral outcome, emphasizing the role of OLs in experience-dependent nervous system plasticity. *Slc16a1* (MCT1) is of particular interest due to its significant role in metabolic support of axons. OL-specific inhibition of MCT1 causes axonal degeneration and motor neuron death, highlighting its potential importance in EE-induced recovery from HX. MCT1 may therefore represent a novel treatment target for premature brain injury. Additionally, other experience-dependent changes in OL-specific mRNAs may be candidates for future therapy, although further studies investigating differential gene expression over time are required before we fully appreciate the role of OLs in neurodevelopment and plasticity, especially after perinatal injury.

In the absence of a definitive treatment for preterm brain injury, EE is a clinically plausible route to enhance functional recovery and attenuate morbid neurodevelopmental insult. Going forward, we must continue to establish and refine interventional strategies, like EE, that promote stimulus-driven gains in functional neurodevelopment. Early intervention, cognitive and behavioral therapy, and comprehensive rehabilitative efforts aimed at motor coordination and balance during critical stages of neurodevelopment would surely benefit recovery. Our findings in a preclinical model of perinatal brain injury offer encouragement. Future studies should investigate how modulation of candidate genes in OLs affects neural circuits, and explore how EE-induced changes drive cognition and behavior on a global scale. Ultimately, strategies implemented in the clinic are likely to depend on a multidisciplinary approach—both pharmacological and environmental interventions that promote cellular maturation and resynchronization to more closely align with normal development.

## Methods

### Animals

Colonies of CNP-EGFP (generated by Dr. V. Gallo, Children’s National Health System, Washington, DC), C57BL/6 (The Jackson Laboratory #003548), wild-type CD1 (Crl:CD1(ICR)); Charles River) and B6: 129-MyRF^tm1Barr^/J (The Jackson Laboratory #010607) mice were maintained at the Children’s National Health System Research Animal Facility. For breeding, heterozygote CNP-EGFP+ males were backcrossed to C57BL/6 females for more than eight generations. In CNP-EGFP mice, various stages of the oligodendrocyte lineage are visualized based on the expression of EGFP driven by a myelin-specific CNP gene promoter^28^. Only mice that expressed EGFP during screening at P2 with ultraviolet goggles were used. To delete MyRF in oligodendrocyte progenitor cells, PDGFRα-CreER^T2^ transgenic mice (The Jackson Laboratory #018280) that had been previously crossed with Rosa26-YFP mice (The Jackson Laboratory #006148) were mated with MyRF^(flox/flox)^ mice (The Jackson Laboratory #010607) to obtain PDGFRα-CreER^T2^: R26R-YFP: MyRF^(flox/flox)^ and MyRF^(+/+)^ littermates. For TRAP-seq experiments, CNP-bacTRAP (The Jackson Laboratory #009159) and PDGFRα-bacTRAP (The Jackson Laboratory #030268) were used. Male and female mice were used in all experiments as preliminary data showed no differences between sexes. All mouse cages (standard and enrichment cages) were individually ventilated. Pups were weaned at P21. All animal procedures were performed ethically, and according to the Institutional Animal Care and Use Committee (IACUC) of the Children’s National Health System (protocol #30473) and the Guide for the Care and Use of Laboratory Animals (National Institutes of Health).

### Hypoxic treatment

Mice were randomly chosen to either undergo hypoxic rearing or serve as normoxic controls. Pups (3d of age; postnatal day 3 (P3)) were exposed to 10−11% oxygen concentration in a hypoxic chamber. To optimize nutrition during hypoxia, transgenic pups were housed in the chamber with two CD1 foster mothers and their pups. At P11, mice were removed from the chamber and transferred to a room with normoxic air conditions. Exposure to hypoxia lasted 8 consecutive days (P3−P11), at which point mouse pups were allocated to the various experimental conditions at the specified time points. This time window in rodent white matter oligodendrocyte development reproduces changes that occur at 23−40 weeks of gestation in the human brain. The hypoxic chamber is a clear, sealed acrylic chamber with two doors at the front allowing access. The interior chamber dimensions are 66″ W × 20″ D × 20″ H. The oxygen content of the chamber is adjusted by a nitrogen/compressed air gas delivery system that mixes the nitrogen with room air using a BioSpherix, Ltd. Pro:OX compact oxygen controller to achieve the desired oxygen levels and moment-to-moment corrections, as needed. The Pro:OX oxygen sensor and instrument were calibrated each week to ensure consistent delivery of appropriate gas levels. Lighting cycles were kept at normal light/dark 12-h cycles.

### Enriched environment

The enriched environment set-up consists of enrichment cages constructed of clear Plexiglas (24 cm W × 20 cm H × 46 cm L). Each cage is equipped with suspended metal link chains or a series of small wooden blocks suspended from the cage roof, a running wheel, a series of clear, colored plastic “habit-trails” of different configurations and several small plastic, hard rubber or wooden balls and objects of different shapes and textures that are scattered over the floor of the cage. Objects are changed, cleaned and rearranged every 3 days during the period of enrichment to maintain novelty. Mice were also provided nesting materials. Mice were reared in larger groups (*n* = 8−12 animals per cage) during periods of enrichment. Standard environment cages were smaller and constructed of clear Plexiglas (16 cm W × 13 cm H × 37 cm L). Mice in these cages were only provided nesting materials and were reared in smaller-sized groups (*n* = 2–5 animals).

### Activity control

To determine the relative individual contribution of locomotor activity, mice were housed in a clear Plexiglas (24 cm W × 20 cm H × 46 cm L) cage containing a running wheel only (without novel enrichment objects) and reared in smaller-sized groups (*n* = 3−5 animals).

### Novel object and socialization control

To determine the relative contributions of socialization and novel object exposure, mice were housed in a clear Plexiglas (24 cm W × 20 cm H × 46 cm L) cage containing novel enrichment objects that were changed, cleaned, and rearranged every 3 days, and reared in larger groups (*n* = 8−12 animals), but without access to a running wheel.

### Behavioral testing

In all behavioral studies, the examiner was blinded to experimental group. Each behavioral experiment was performed using naïve mice that had not undergone any previous experimental testing. The qualitative impact of EE on functional recovery in developing WM was evaluated using the inclined beam-walking task. This test evaluates WM-specific behavioral deficits and recovery between experimental groups and controls. The inclined beam task was performed as follows: two elevated 80-cm-long wooden beams were placed at a 30° angle. One beam was 2 cm wide, and the other was 1 cm wide. A dark box with animal bedding was placed at the top of the inclined beam as a target for each mouse to reach. The beam was cleaned with 30% ethanol between each mouse. The number of foot slips was documented and averaged during three or four trials for each mouse. Analysis was performed at P45.

### Tissue processing and immunohistochemistry

Immunohistochemical analysis was performed at various time points throughout development. In each experiment, normoxic mice served as controls. Hypoxic or normoxic mice were anesthetized with isoflurane and transcardially perfused with 0.1 M phosphate-buffered saline (PBS), pH 7.4, followed by 4% paraformaldehyde (PFA). Brains were postfixed in 4% PFA overnight, and coronally sectioned at 40 μm with a sliding microtome, collected in PBS, pH 7.4, and stored at 4 °C until use.

Immunohistochemistry was performed on free-floating sections, which were blocked for 1 h in a solution containing PBST (0.1% TritonX-100) and 20% normal goat serum (NGS), followed by overnight incubation at 4 °C in primary antibodies diluted in PBST and 5% NGS. Primary antibody dilutions were 1:500 for rat anti-BrdU (Abcam), rabbit anti-GFAP (Abcam), mouse anti-SMI31 (Biolegend), mouse anti-SMI32 (Biolegend), rabbit anti-IBA1 (Wako), rabbit anti-CD68 (Abcam), and 1:250 for rabbit anti-NG2 (Millipore), rabbit anti-Olig2 (Millipore), rabbit anti-Ki67 (Abcam), and mouse anti-CC1 (Calbiochem), rat anti-PDGFRɑ (BD Biosciences), rabbit anti-cleaved caspase 3 (Cell Signaling). Sections were incubated with species appropriate secondary antibodies (1:500) for 2 h at RT. Secondary antibodies included: Alexa Fluor 647—donkey anti-mouse (Jackson Immunoresearch; 715-605-150), Alexa Fluor 594—donkey anti-rabbit (Jackson Immunoresearch; 711-585-152), and Alexa Fluor 647 donkey anti-rat (Jackson Immunoresearch; 712-605-150). Sections were treated with 4′, 6-diamidino-2-phenylindole (DAPI) for 10−15 min and mounted with Mowiol.

### In situ hybridization (ISH)

ISH was performed on C57bl/6n brain sections (16 µm) as previously described^75^. Briefly, DIG-labeled probes were generated by PCR using cDNA generated from P14 C57bl/6n whole-brain RNA. Sections were postfixed with 4% PFA for 10 min, followed by three, 5-min washes in PBS, and a 10 min incubation in acetylation mix (0.1% triethanolamine and 0.02% acetic anhydride in water). After three more PBS washes, sections were incubated in warm hybridization buffer (50% formamide, 5× saline sodium citrate (SSC), 5× Denhardts, 250 µg/ml yeast RNA, 500 µg/ml salmon sperm in water) for an hour at 68 °C, followed by an overnight incubation with 500 ng/ml probe in hybridization buffer. Next, sections had sequential hour-long incubations (68 °C) in formamide solution #1 (%50 formamide, 5× SSC, 1% sodium dodecyl sulfate (SDS) in water) and formamide solution #2 (%50 formamide, 2× SSC, 1% Tween20 in water). Following two, 10-min washes with 1% Tween20 in 1× Tris-buffered saline (TBST), sections were blocked with 10% heat-inactivated horse serum in TBST for 1 h at room temperature. Sections were then incubated with anti-DIG antibody (1:2000) for 1.5 h at room temperature, before development with 0.02% NBT-BCIP (Roche) in alkaline phosphatase buffer.

### Image acquisition and analysis

We used a confocal LSM (Zeiss 510) microscope using ×40 oil objectives to image CNP-EGFP tissue. Z-stack images of 1-μm-thick single planes were captured using Zeiss software. Four different lasers were used to image localization of FITC (488 nm excitation), CY3 (580 nm excitation), CY5 (647 nm excitation), and DAPI (400 nm excitation). A Zeiss ApoTome.2 microscope was also used for optical sectioning of PDGFRα-CreER^T2^: R26R-YFP: MyRF^(flox/flox)^ and MyRF^(+/+)^ tissue. The images were acquired using Plan-NeoFluar×40/1.03 oil objective. The Colibri 7 Type RGB-UV light source equipped with four solid-state LED lines 385, 475, 555 and 630 nm were used to excite the fluorochromes and the fluorescence was collected on to the Axiocam 503 monochromatic camera through filter Set 90 HE (Excitation filters BP 385/30, BP 469/38, BP555/30, BP 631/33; QBS 405 + 493 + 575 + 653; Emission filters QBP 425/30). ZEN 2.3 (blue edition) software was used to acquire the images.

Images were viewed using NIH ImageJ and the Zeiss LSM Image Browser. All histological quantifications were performed in a blinded manner. Immunolabeled cells were manually counted in each optical section using the ImageJ “Cell Counter” plugin. Data were obtained from three to eight mice per experimental group. An average of six bilateral images were taken for each section of white matter, including two from the corpus callosum, two from the cingulum, and two from the external capsule region. For each image, the total number of cells was counted and normalized to volume.

### Electron microscopy

Mice were prepared for standard transmission electron microscopic analysis as previously described^76^. Briefly, deeply anesthetized mice at age P45 were transcardially perfused with 0.1 M Millonig’s Phosphate Buffer containing 4% PFA and 5% gluteraldehyde. Following two weeks of aldehyde postfixation, brains were harvested, grossly cut into 1 mm sagittal sections using a brain matrix, thoroughly rinsed in 0.1 M cacodylate buffer, postfixed in 2% osmium tetroxide, rinsed in 0.1 M cacodylate buffer, dehydrated in serial dilutions of ethanol, and embedded in Polybed 812 resin (PolySciences, Warrington, PA). One-micron sections were stained with toluidine blue and used to identify the body of the corpus callosum at the level of the fornix. Ninety nanometer sections of this region were stained with uranyl acetate and lead citrate. Sagittal sections of white matter were examined with a JEOL JEM 1400 plus transmission electron microscope (JEOL, Peabody, MA) equipped with a Gatan One View 4 K CCD camera (Gatan Inc., Pleasanton, CA) housed in the Department of Anatomy and Neurobiology Microscopy and Imaging Facility at Virginia Commonwealth University. For each sample, 15 electron micrographs were collected from the corpus callosum and used for myelin thickness (*g*-ratio analysis). Measurements and image processing were performed using NIH ImageJ software. Two axon diameters (the longest and shortest), and two myelin widths (the widest and thinnest) were measured for each myelinated axon. Myelin thickness was calculated from the average of radial measurements at four points per sheath, avoiding areas of tongue processes or fixation artifact. The extent of myelination was quantitatively compared by determining the *g*-ratios, which were calculated by dividing the diameter of the axon by the diameter of the entire myelinated fiber. The *g*-ratios were plotted against axon diameter using a scatter plot to compare myelin thickness and axon caliber between experimental groups. Axon diameters were calculated from measurement of the axon circumference. Axons with diameters typical of unmyelinated fibers (<0.3 μm) were excluded from analysis. Measurements were performed blinded to experimental groups, and at least 100 axons were measured for each brain.

### Western blot analysis

Protein quantification analysis was conducted on microdissected white matter tissue that included the corpus callosum, cingulum, and external capsule. Subcortical white matter was dissected on ice-cold medium from 300-μm coronal sections from animals in each experimental group at P45. Only sections anterior to the hippocampus were used for dissection. The dissected white matter was rinsed with ice-cold PBS, and homogenized on ice in 150–200 μl of RIPA lysis buffer with protease inhibitors (Santa Cruz Biotechnology). Protein concentrations were determined by using the Bradford protein assay kit (Bio-Rad). Western blot analysis was performed using 10–20 μg of total cell lysates. Protein extracts were boiled for 5 min before loading onto 4–20% gradient Tris glycine gels (NuSep). Gels were electrotransferred to a 0.2 μm nitrocellulose polyvinylidene fluoride (PVDF) membrane (Millipore) in transfer buffer overnight at 4 °C. Membranes were blocked in 5% milk in Tris-buffered saline-Tween (TBST) for 1 h, then incubated at 4 °C overnight with one of the following primary antibodies diluted in 1% bovine serum albumin (Sigma Aldrich): mouse anti-MBP (1:5000, Covance), mouse anti-Actin (1:5000, Millipore), and rabbit anti-MAG (1:500, Santa Cruz Biotechnology). The membranes were then washed in TBST three times for 10–15 min at room temperature, followed by the addition of species-specific horseradish-peroxidase-conjugated goat anti-rabbit (Santa Cruz) or goat anti-mouse (Santa Cruz) secondary antibodies (1:5000) diluted in 5% milk in TBST for 3 h. Chemiluminescent signals were detected with ECL substrate (GE Healthcare) and visualized by X-ray exposure. Band intensity was measured using ImageJ software. Western blots were obtained from the white matter of three mice in each experimental group and time point. Data were averaged and represented as means ± SEM. Full scan images of the blots are provided in the source data file.

### BrdU injections

To evaluate 5-bromo-2′-deoxyuridine (BrdU, Sigma) incorporation after HX injury, mice were subjected to cumulative (pulse) intraperitoneal (10 mg/kg) BrdU injections once every 24 h from P11 to P15. At P45, mice were sacrificed, transcardially perfused, and tissue was fixed and analyzed using immunohistochemical techniques. To evaluate BrdU incorporation during environmental enrichment, mice were subjected to cumulative (pulse) intraperitoneal (10 mg/kg) BrdU injections once every 24 h from P24 to P28. At P45, mice were sacrificed, transcardially perfused, and tissue was fixed and analyzed using immunohistochemical techniques.

### Tamoxifen injections

Tamoxifen (Sigma) was dissolved in 100% ethanol and then diluted in autoclaved sunflower oil (Sigma) to a final concentration of 10 mg/ml. PDGFRα-CreER^T2^: R26R-YFP: MyRF^(flox/flox)^ and MyRF^(+/+)^ littermates were injected intraperitoneally with 75 mg/kg tamoxifen once per day from P13 to P17.

### Quantitative PCR

Total RNA was isolated from microdissected white matter using the RNeasy lipid tissue mini kit (Qiagen). Synthesis of cDNA was carried out using the iScript Reverse Transcription Supermix for RT-qPCR (Bio-Rad). qPCR was performed on a CFX96 real-time system (Bio-Rad) in a 20-μl reaction mixture using SsoAdvanced Universal SYBR Green PCR master mix (Bio-Rad). Cycle parameters were 3 s at 95 °C and 30 s at 60 °C. Data were normalized to GAPDH. *MyRF* primers: 5′-CCAGAAGAAGAACCACTTCCA-3′ and 5′-CACCATGCAGCTTCAGATAGA-3′. *Gapdh* primers: 5′-CTTTGTCAAGCTCATTTCCTGG-3′ and 5′-TCTTGCTCAGTGTCCTTGC-3′.

### Hippocampus analysis

Control experiments evaluating continuous environmental enrichment (P15−P45) vs. a nonenriched environment were performed under normoxic conditions. Analysis was performed at P45. The total number of DAPI, Ki67, Nestin, Sox2, and Dcx-positive cells in the dentate gyrus were determined in 5−7 coronal sections collected 240 µm apart. All positive cells were counted in the granular cell layer and subgranular layer on 40-µm-thick sections. The subgranular zone (SGZ) was defined as a one to two cell diameter-wide zone from the hilus border toward the granular layer. Cell quantification was done on a confocal LSM microscope (Zeiss 510) under 40×, 1 µm step, on 40-µm-thick immunostained tissue sections with a volume of 225 µm × 225 µm × 10 µm (*x*, *y*, *z*). The analysis was limited to the boundaries of the dentate gyrus, as defined by DAPI distribution. Results are expressed as mean ± SEM. Unpaired *t* test (two-tailed) was performed to establish statistical significance.

### Translating ribosome affinity purification

RNA was isolated from PDGFRα-bacTRAP or CNP-bacTRAP mice (*n* *=* 3−7 per group) using a modified version of the previously described protocol^77^. Briefly, subcortical white matter was rapidly dissected in ice-cold dissection buffer (2.5 mM  4-(2-hydroxyethyl)-1-piperazineethanesulfonic acid  (HEPES), 35 mM glucose and 4 mM NaHCO_3_, and 100 μg/ml cycloheximide in Hank’s balanced salt solution (HBSS)), homogenized in lysis buffer (20 mM HEPES, 150 mM KCl and 10 mM MgCl_2_, 0.5 mM dithiothreitol (DTT), 100 μg/ml cycloheximide, 10 μl/ml rRNasin, 10 μl/ml Superasin, and ethylenediaminetetraacetic acid (EDTA)-free protease inhibitors in RNase-free water), and then centrifuged at 1000 × *g* for 10 min. 1% NP-40 and 1% DHPC were added to supernatants, chilled on ice for 5 min, and centrifuged at 10,000 × *g* for 10 min. Supernatants incubated overnight at 4 °C with GFP antibodies (HtzGFP-19F7 and HtzGFP-19C8, Memorial Sloan Kettering Center) bound to Streptavidin MyOne T1 Dynabeads (Invitrogen). Beads were washed four times in a high salt buffer (20 mM HEPES, 350 mM KCl, 10 mM MgCl_2_, 1% NP-40, 0.5 mM DTT, and 100 μg/ml cycloheximide in RNase-free water), and RNA was isolated using the Absolutely RNA Nanoprep Kit (Agilent).

### RNA-sequencing

RNA samples were sent to the Genome Sciences and Bioinformatics core at Penn State College of Medicine for Library preparation and RNA-sequencing. RNA quality was determined using the Bioanalyzer Pico Kit (Agilent), and quantity was determined with the Qubit fluorometer (ThermoFisher). Libraries were prepared from RNA (RIN > 7) using the Trio RNA-Seq Library Preparation Kit (NuGen), according to the manufacturer’s protocol. Libraries were sequenced using the NovaSeq 6000 System (Illumina) to obtain 50 base pair (bp), paired-end reads. The first 5 bp were trimmed from the 5′ end of the reads using Trimmomatic^78^, as recommended by the Library Preparation Kit. Reads were mapped to the mouse reference genome (Genome Reference Consortium Mouse Build 38 – mm10) using STAR^79^, and read counts were generated using HTseq^80^. Differential gene expression and normalized read counts were generated using DESeq2 package for R^81^. DEGs reported have normalized read counts above 5 in all samples, and an adjusted *p* < 0.05 (Wald test with Benjamini−Hochberg post hoc). Ingenuity Pathway Analysis (Qiagen) was used to identify cellular and molecular functions and the associated genes implicated from DEGs between the HX and HX-EE groups.

### Statistical analysis

Specific numbers of animals are denoted in each figure legend. Data were compiled and organized using Microsoft Excel. Prior to statistical testing, D’Agostino & Pearson normality test was used to show that our data were normally distributed. Significance was calculated using GraphPad Prism 7.0 software. All data are presented as averages ± SEM. All cell counting, western blot, and behavioral data were statistically compared using one-way ANOVA to determine whether overall differences exist across study groups at specific ages. Comparisons between groups were treated as unplanned comparisons, which were adjusted using a Tukey’s correction. A two-tailed type 1 error (*p* value < 0.05) was used to determine statistical significance. The degree of statistical significance was denoted using asterisks (*p < 0.05; **p < 0.01; ***p < 0.005). Where relevant, exact *p* values are provided.

### Reporting summary

Further information on research design is available in the Nature Research Reporting Summary linked to this article.

## Supplementary information

Supplementary Information

Peer Review

Reporting Summary

Description of Additional Supplementary Files

Supplementary Dataset 1

Supplementary Dataset 2

Supplementary Dataset 3

Supplementary Dataset 4

Supplementary Dataset 5

Supplementary Dataset 6

Supplementary Dataset 7

Supplementary Dataset 8

Supplementary Dataset 9

Supplementary Dataset 10

Supplementary Dataset 11

Supplementary Dataset 12

## Data Availability

All RNA-sequencing data have been uploaded to the Sequence Read Archive (NCBI). Accession code: PRJNA597018. All other data is available in the source file.

## Source data

Source Data
