# Supplementary material for: Environmental enrichment ameliorates perinatal brain injury and promotes functional white matter recovery
Source: Nat Commun. 2020 Feb 19;11:964. doi: 10.1038/s41467-020-14762-7 (PMC7031237; doi:10.1038/s41467-020-14762-7)
Supplement: Supplementary file 1 — Supplementary Information [file 41467_2020_14762_MOESM1_ESM.pdf]

Supplementary Information for:

# Environmental enrichment ameliorates perinatal brain injury and promotes functional white matter recovery

Thomas A. Forbes[1,2]*, Evan Z. Goldstein[1]*, Jeffrey L. Dupree[3], Beata Jablonska[1,2], Joseph Scafidi[1,2], Katrina L. Adams[1], Yuka Imamura[4], Kazue Hashimoto-Torii[1], and Vittorio Gallo[1,2,†]

* These authors contributed equally

†Correspondence to: vgallo@cnmcresearch.org (V. Gallo).

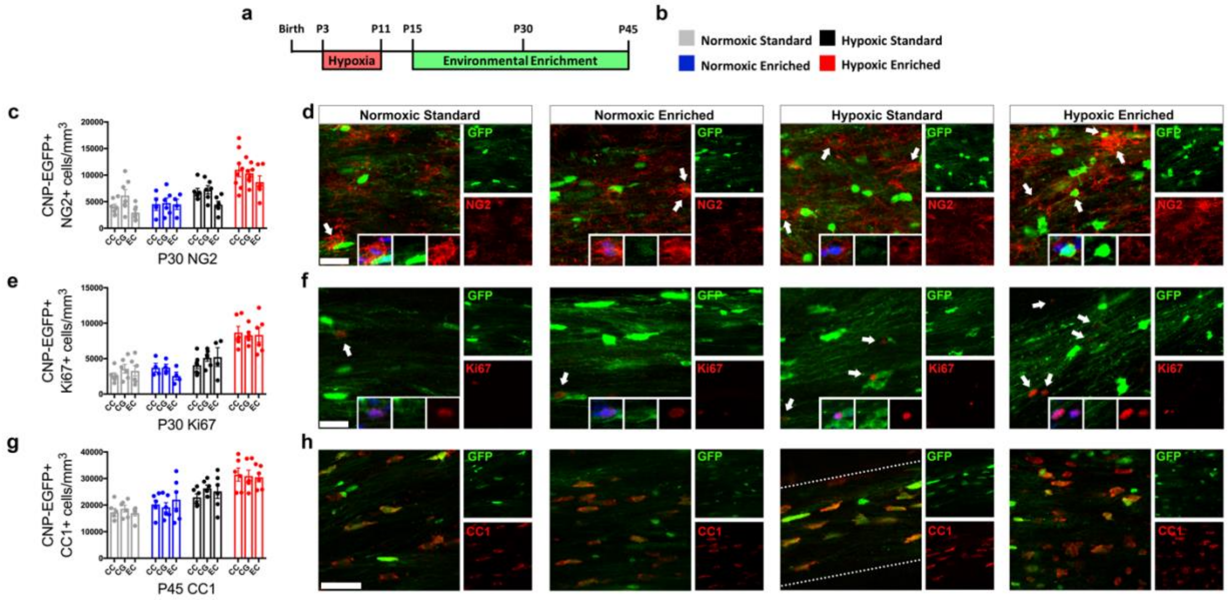


**Supplementary Figure 1. Region-specific breakdown and immunohistochemical labeling of OL lineage and proliferation markers from the subcortical WM of CNP-EGFP mice. (a)** Experimental paradigm**. (b)** Experimental groups**. (c)** WM region-specific quantification of EGFP+NG2+ OPCs at P30 (CC = corpus callosum, CG = cingulum, EC = external capsule) (NX, *n* = 7; NX-EE, *n* = 6; HX, *n* = 6; HX-EE, *n* = 8). **(d)** Representative images of EGFP- (green) and NG2- (red) expressing OPCs for all groups. Green fluorescence is much reduced in OPCs compared with OL. Arrows and insets are provided to assist in the identification of co-labeled cells (DAPI labeled in blue is included in inset images). Scale bar = 30μm. **(e)** WM region-specific quantification of EGFP+Ki67+ proliferating OPCs at P30 (NX, *n* = 6; NX-EE, *n* = 5; HX, *n* = 7; HX-EE, *n* = 6). **(f)** Representative images of EGFP- (green) and Ki67- (red) expressing proliferating OPCs. Scale bar = 30μm. **(g)** WM region-specific quantification of EGFP+CC1+ OLs at P45 (*n* = 6 for all groups) **(h)** Representative images of EGFP- (green) and CC1- (red) expressing OLs. Scale bar = 50μm. Dotted white lines illustrate the WM/GM border. Data expressed as mean ± SEM. Source data are provided as a Source Data file.

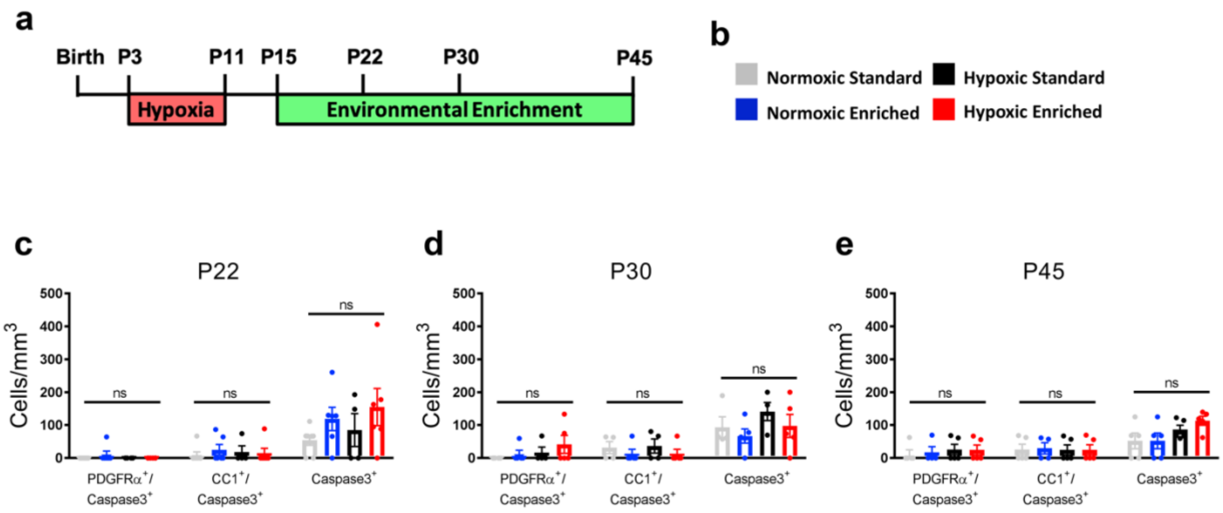


**Supplementary Figure 2. Environmental enrichment has no effect on caspase3-mediated apoptosis in the subcortical white matter. (a)** Experimental paradigm**. (b)** Experimental groups**. (c-e)** Quantification of PDGFRα+Cleaved Caspase3+, CC1+Cleaved Caspase3+, or total Cleaved Caspase3+ cells at P22 (NX, *n* =7; NX-EE, *n* = 6; HX, *n* = 4; HX-EE, *n* = 6) **(c)**, P30 (NX, *n* =4; NX-EE, *n* = 5; HX, *n* = 4; HX-EE, *n* = 5) **(d)**, and P45 (NX, *n* =5; NX-EE, *n* = 4; HX, *n* = 5; HX-EE, *n* = 5) **(e)**. Data expressed as mean ± SEM. One-way ANOVA followed by a Tukey's post hoc test for multiple comparisons was used to compare differences between groups. Source data are provided as a Source Data file.

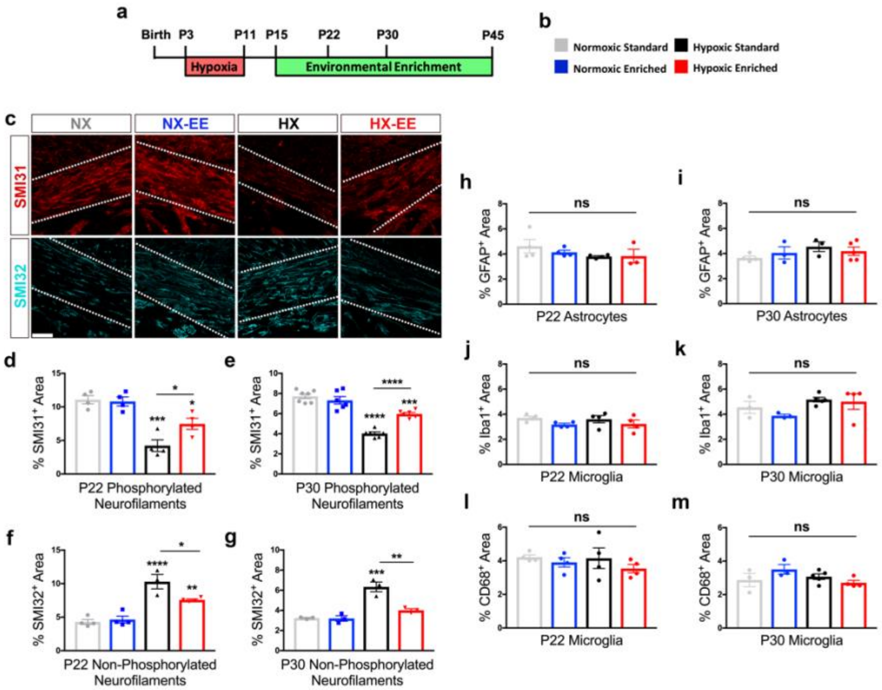

**Supplementary Figure 3. Other cell types involved in environmental enrichment-induced recovery from perinatal brain injury. (a)** Experimental paradigm**. (b)** Experimental groups**. (c)** Representative images of phosphorylated neurofilaments (SMI31, red) and non-phosphorylated neurofilaments (SMI32, blue) in the subcortical white matter for all groups. Dotted white lines illustrate the WM/GM border. Scale bar = 50µm. **(d-e)** Quantification of phosphorylated neurofilament immunofluorescence (% SMI31+ area) at P22 (*n* = 4 per group) (*** *p* = 0.0002, * *p* = 0.024, * *p* = 0.044) **(d)** and P30 (NX, *n* =7; NX-EE, *n* = 6; HX, *n* = 6; HX-EE, *n* = 6) (*** *p* = 0.0002, **** *p* < 0.0001) **(e)**. **(f-g)** Quantification of non-phosphorylated neurofilament immunofluorescence (% SMI32+ area) at P22 (NX, *n* =4; NX-EE, *n* = 4; HX, *n* = 3; HX-EE, *n* = 4) (**** *p* < 0.0001, ** *p* = 0.004, * *p* = 0.025) **(f)** and P30 (*n* = 3 per group) (*** *p* = 0.0003, ** *p* = 0.002) **(g)**. **(h-i)** Quantification of astrocyte immunofluorescence (% GFAP+ area) at P22 (NX, *n* =4; NX-EE, *n* = 4; HX, *n* = 4; HX-EE, *n* = 3) **(h)** and P30 (NX, *n* =4; NX-EE, *n* = 3; HX, *n* = 3; HX-EE, *n* = 5) **(i)**. **(j-k)** Quantification of microglia immunofluorescence (% IBA1+ area) at P22 (*n* = 4 per group) **(j)** and P30 (NX, *n* =3; NX-EE, *n* = 3; HX, *n* = 5; HX-EE, *n* = 4) **(k)**. **(l-m)** Quantification of phagocytic microglia immunofluorescence (% CD68+ area) at P22 (*n* = 4 per group) **(l)** and P30 (NX, *n* =3; NX-EE, *n* = 3; HX, *n* = 5; HX-EE, *n* = 4) **(m)**. Data expressed as mean ± SEM. One-way ANOVA followed by a Tukey's post hoc test for multiple comparisons was used to compare differences between groups. Source data are provided as a Source Data file.

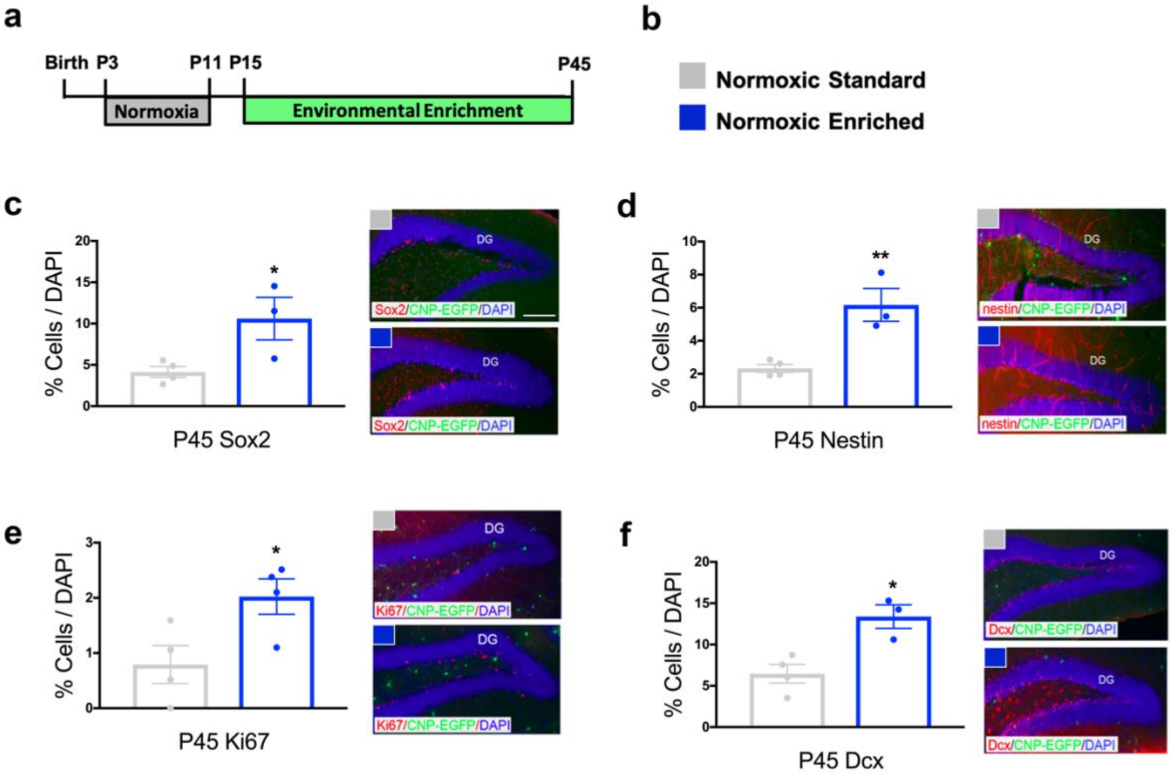


**Supplementary Figure 4. Environmental enrichment promotes hippocampal stem cell proliferation and neurogenesis under normal physiological conditions (NX). (a)** Experimental paradigm**. (b)** Experimental groups**. (c-f)** Quantification of Sox2+ cells (NX, *n* = 4; NX-EE, *n* = 3; * *p = 0.03*) **(c)**, Nestin+ cells (NX, *n* = 4; NX-EE, *n* = 3; ** *p = 0.007*). Scale bar = 100μm. **(d)**, Ki67+ cells (NX, *n* = 4; NX-EE, *n* = 4; * *p = 0.03*) **(e)**, and Dcx+ cells (NX, *n* = 4; NX-EE, *n* = 3; * *p = 0.01*) **(f)** in the dentate gyrus of the hippocampus at P45. Representative images for each cell population are shown. Data are expressed as the mean ± SEM percentage of cells per total DAPI labeled cells. A two-tailed, unpaired t-test was used to compare differences for each marker. Source data are provided as a Source Data file.

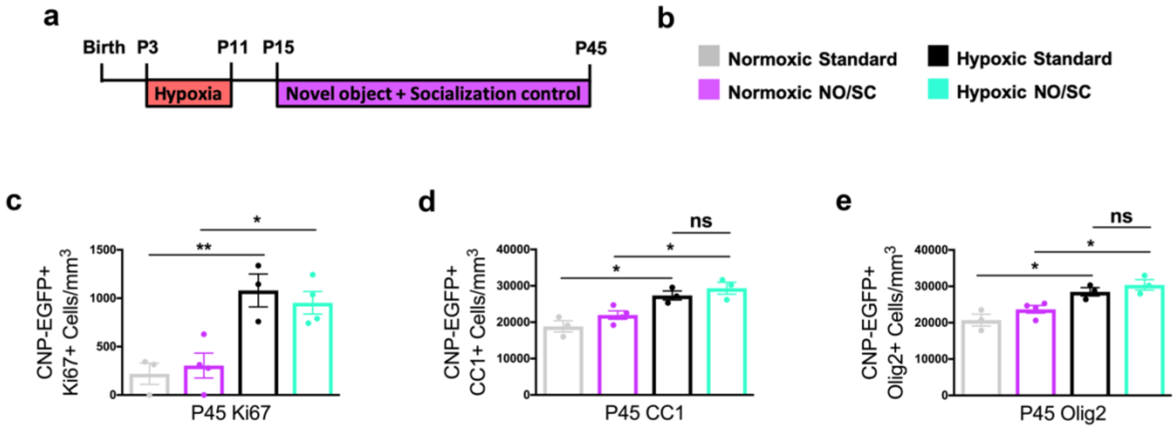


**Supplementary Figure 5. Novel object exposure and increased socialization has no effect on OL recovery after perinatal brain injury. (a)** Experimental paradigm. Mice were placed in large social groups and exposed to novel objects, without access to a running wheel. **(b)** Experimental groups (NX, NX Novel Object and Socialization Control (NX-NO/SC), HX, HX Novel Object and Socialization Control (HX-NO/SC). **(c)** Quantification of EGFP+Ki67+ cells at P45 (* $p = 0.017$; ** $p = 0.007$) (NX, $n = 3$; NX-NO/SC, $n = 4$; HX, $n = 3$; HX NO/SC, $n = 4$). **(d)** Quantification of EGFP+CC1+ OLs at P45 (From left to right: * $p = 0.011$, * $p = 0.016$, ns = not significant) (NX, $n = 3$; NX-NO/SC, $n = 4$; HX, $n = 3$; HX-NO/SC, $n = 3$). **(e)** Quantification of EGFP+Olig2+ cells at P45 (From left to right: * $p = 0.012$, * $p = 0.019$) (NX, $n = 3$; NX-NO/SC, $n = 4$; HX, $n = 3$; HX-NO/SC, $n = 3$). Data expressed as mean ± SEM. One-way ANOVA followed by a Tukey's post hoc test for multiple comparisons was used to compare differences between groups. Source data are provided as a Source Data file.

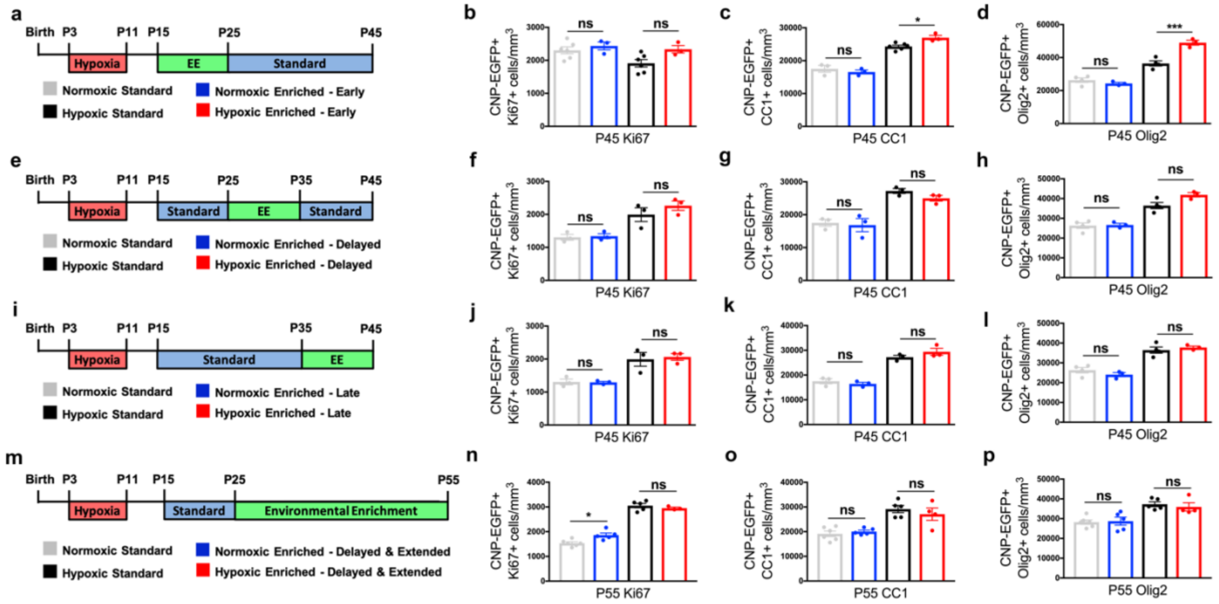


**Supplementary Figure 6. Alternative paradigms of non-continuous environmental enrichment did not improve WM recovery after perinatal brain injury. (a)** Early intervention paradigm exposed mice to EE from P15-P25 only. **(b)** Quantification of EGFP+Ki67+ OPCs at P45 following early EE (NX, $n$ = 6; NX-EE, $n$ = 3; HX, $n$ = 6; HX-EE, $n$ = 3). **(c)** EGFP+CC1+ OLs at P45 following early EE (NX, $n$ = 3; NX-EE, $n$ = 3; HX, $n$ = 6; HX-EE, $n$ = 3) (* $p$ = 0.04). **(d)** EGFP+Olig2+ cells following early EE (NX, $n$ = 4; NX-EE, $n$ = 3; HX, $n$ = 4; HX-EE, $n$ = 3) (*** $p$ = 0.0004). **(e)** Delayed intervention paradigm: mice were exposed to EE from P25-P35. **(f)** Quantification of EGFP+Ki67+ OPCs following delayed EE ($n$ = 3 per group). **(g)** EGFP+CC1+ OLs following delayed EE ($n$ = 3 per group). **(h)** EGFP+Olig2+ cells at P45 following delayed EE (NX, $n$ = 4 animals; NX-EE, $n$ = 3 animals; HX, $n$ = 4 animals; HX-EE, $n$ = 3 animals). **(i)** Late intervention paradigm exposed mice to EE from P35 to P45. **(j)** Quantification of EGFP+Ki67+ OPCs following late EE ($n$ = 3 per group). **(k)** EGFP+CC1+ OLs following late EE ($n$ = 3 per group). **(l)** EGFP+Olig2+ cells at P45 following late EE ($n$ = 3 per group). **(m)** Delayed and extended intervention paradigm exposed mice to EE from P25-P55. **(n)** Quantification of EGFP+Ki67+ OPCs at P45 following early EE (NX, $n$ = 6; NX-EE, $n$ = 5; HX, $n$ = 5; HX-EE, $n$ = 3) (* $p$ = 0.02). **(o)** EGFP+CC1+ OLs at P45 following early EE (NX, $n$ = 6; NX-EE, $n$ = 5; HX, $n$ = 5; HX-EE, $n$ = 4). **(p)** EGFP+Olig2+ cells following early EE (NX, $n$ = 6; NX-EE, $n$ = 5; HX, $n$ = 5; HX-EE, $n$ = 4). Data expressed as mean ± SEM. One-way ANOVA followed by a Tukey's post hoc test for multiple comparisons was used to compare differences between groups. Source data are provided as a Source Data file.

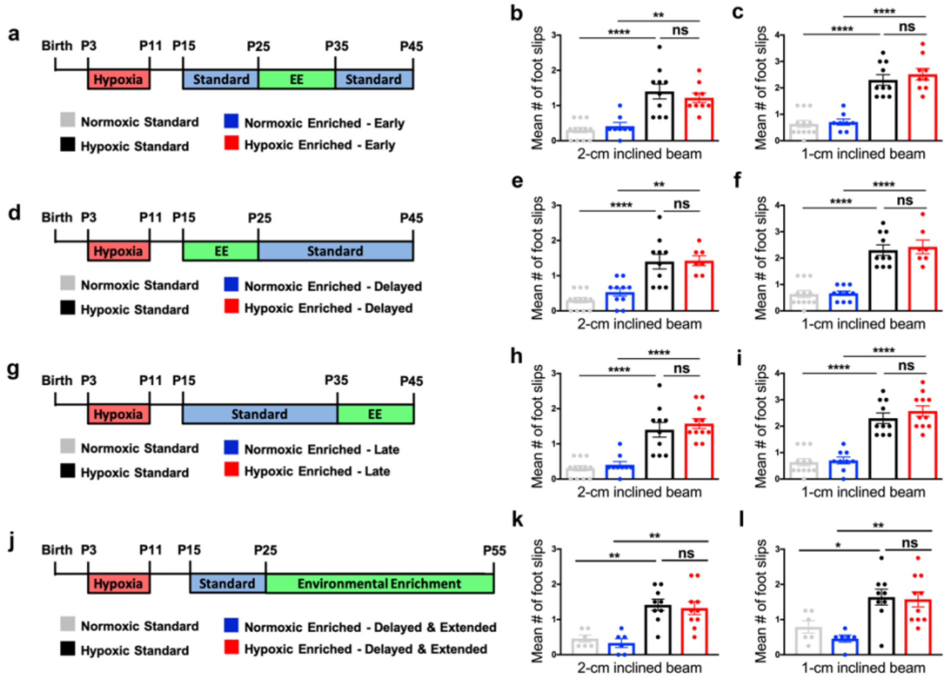


**Supplementary Figure 7. Number of foot slips on inclined beam task for alternative paradigms of environmental enrichment. (a)** Early intervention paradigm exposed mice to EE from P15-P25 only. **(b-c)** Quantification of foot slips on 2-cm inclined beam (**** $p < 0.0001$, ** $p = 0.003$) **(b)** and 1-cm inclined beam (**** $p < 0.0001$) **(c)** at P45 following early EE (NX, $n = 12$; NX-EE, $n = 8$; HX, $n = 10$; HX-EE, $n = 9$). **(d)** Delayed intervention paradigm: mice were exposed to EE from P25-P35. **(e-f)** Quantification of foot slips on 2-cm inclined beam (**** $p < 0.0001$, ** $p = 0.001$) **(e)** and 1-cm inclined beam (**** $p < 0.0001$) **(f)** at P45 following delayed EE (NX, $n = 12$; NX-EE, $n = 10$; HX, $n = 10$; HX-EE, $n = 7$). **(g)** Late intervention paradigm exposed mice to EE from P35 to P45. **(h-i)** Quantification of foot slips on 2-cm inclined beam (**** $p < 0.0001$) **(h)** and 1-cm inclined beam (**** $p < 0.0001$) **(i)** at P45 following late EE (NX, $n = 12$; NX-EE, $n = 9$; HX, $n = 10$; HX-EE, $n = 11$). **(j)** Delayed and extended intervention paradigm exposed mice to EE from P25-P55. **(k-l)** Quantification of foot slips on 2-cm inclined beam (From left to right: ** $p = 0.003$, ** $p = 0.001$) **(k)** and 1-cm inclined beam (* $p = 0.05$, ** $p = 0.005$) **(l)** at P45 following early EE (NX, $n = 6$; NX-EE, $n = 6$; HX, $n = 9$; HX-EE, $n = 10$). Data expressed as mean ± SEM. One-way ANOVA followed by a Tukey's post hoc test for multiple comparisons was used to compare differences between groups. Source data are provided as a Source Data file.

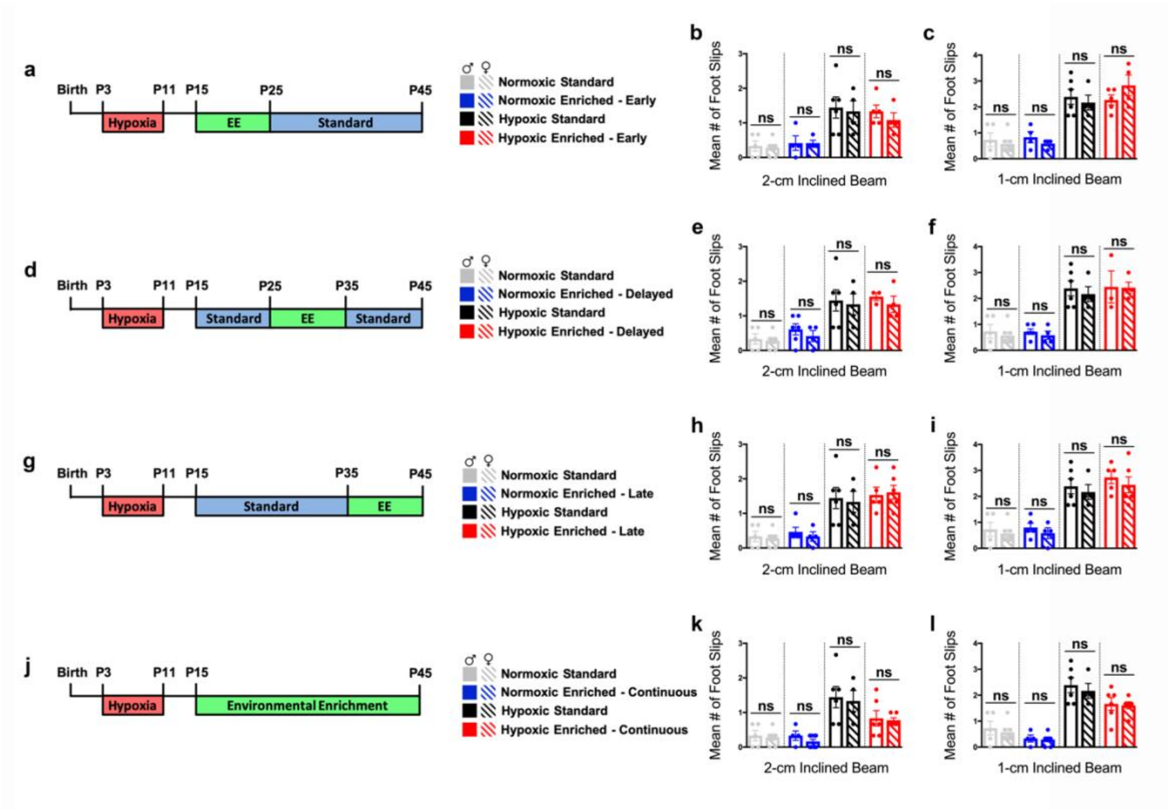


**Supplementary Figure 8. Sex comparison of inclined beam task for alternative paradigms of environmental enrichment. (a)** Early EE intervention paradigm. **(b-c)** Quantification of foot slips on 2-cm inclined beam **(b)** and 1-cm inclined beam **(c)** following early EE, with males and females analyzed separately (NX, *n* = 12 (7 females, 5 males); NX-EE, *n* = 8 (4 females, 4 males); HX, *n* = 10 (4 females, 6 males); HX-EE, *n* = 9 (4 females, 5 males)). **(d)** Delayed EE intervention paradigm. **(e-f)** Quantification of foot slips on 2-cm inclined beam **(e)** and 1-cm inclined beam **(f)** following delayed EE, with males and females analyzed separately (NX, *n* = 12 (7 females, 5 males); NX-EE, *n* = 10 (4 females, 6 males); HX, *n* = 10 (4 females, 6 males); HX-EE, *n* = 7 (4 females, 3 males)). **(g)** Late EE intervention paradigm. **(h-i)** Quantification of foot slips on 2-cm inclined beam **(h)** and 1-cm inclined beam **(i)** following late EE, with males and females analyzed separately (NX, *n* = 12 (7 females, 5 males); NX-EE, *n* = 9 (4 females, 5 males); HX, *n* = 10 (4 females, 6 males); HX-EE, *n* = 11 (6 females, 5 males)). **(j)** Continuous EE intervention paradigm. **(k-l)** Quantification of foot slips on 2-cm inclined beam **(k)** and 1-cm inclined beam **(l)** following continuous EE, with males and females analyzed separately (NX, n = 12 (7 females, 5 males); NX-EE, n = 12 (8 females, 4 males); HX, n = 10 (4 females, 6 males); HX-EE, n = 12 (6 females, 6 males)). Data expressed as mean ± SEM. One-way ANOVA followed by a Tukey's post hoc test for multiple comparisons was used to compare differences between groups. Source data are provided as a Source Data file.

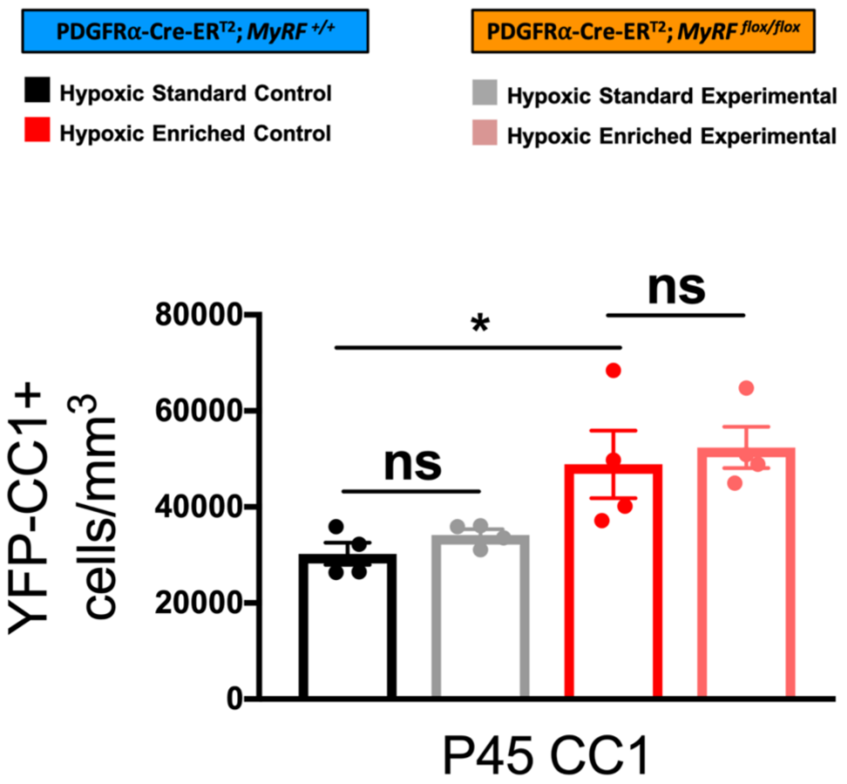


**Supplementary Figure 9. Quantification of total YFP(-) CC1(+) OLs at P45.** $n$ = 4 mice per group. * $p = 0.044$; ns = not significant. Data expressed as mean ± SEM. One-way ANOVA followed by a Tukey's post hoc test for multiple comparisons was used to compare differences between groups. Source data are provided as a Source Data file.

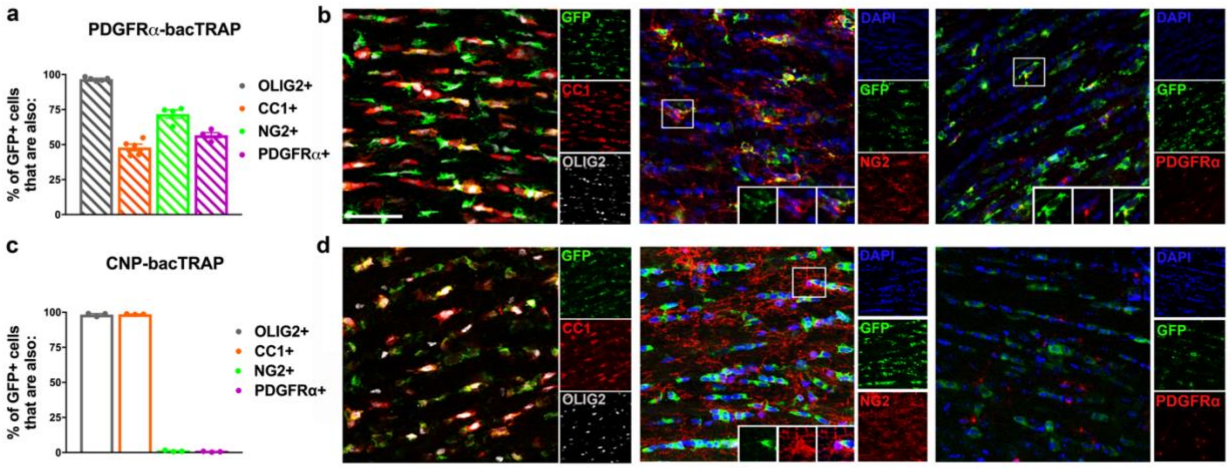


**Supplementary Figure 10. Characterization of oligodendrocyte lineage cells in PDGFRα-bacTRAP and CNP-bacTRAP mice. (a)** Characterization of *PDGFRα-bacTRAP* mouse subcortical white matter. Data represent the mean ± SEM of GFP-expressing cells that also express: Olig2 (OL-lineage cells; *n* = 5), CC1 (OLs; *n* = 5), NG2 (OPCs; *n* = 4), or PDGFRα (OPCs; *n* = 4). **(b)** Representative images of corpus callosum from *PDGFRα-bacTRAP* mice immunohistochemically labeled for: GFP (PDGFRα$^{+}$-OPC ribosomes; green), CC1 (red), and Olig2 (white), DAPI (nuclei; blue), GFP (green), and NG2 (red), or DAPI (nuclei; blue), GFP (green), and PDGFRα (red). Inset images show a magnified view of GFP colocalization with NG2 or PDGFRα-expressing cells. Immunohistochemical labeling was performed once using established protocols. **(c)** Characterization of *CNP-bacTRAP* mouse subcortical white matter. Data represent the mean ± SEM of GFP-expressing cells that also express: OLIG2 (OL-lineage cells), CC1 (OLs), NG2 (OPCs), or PDGFRα (OPCs; *n* = 3 mice). **(d)** Representative images of corpus callosum from *CNP-bacTRAP* mice immunohistochemically labeled for: GFP (CNP$^{+}$-oligodendrocyte ribosomes; green), CC1 (red), and OLIG2 (white), DAPI (nuclei; blue), GFP (green), and NG2 (red), or DAPI (nuclei; blue), GFP (green), and PDGFRα (red). Inset images show a magnified view of an NG2-expressing cell, juxtaposed to (but not co-localized to) a GFP-expressing cell. Immunohistochemical labeling was performed once using established protocols. Scale bar = 50μm. Source data are provided as a Source Data file.

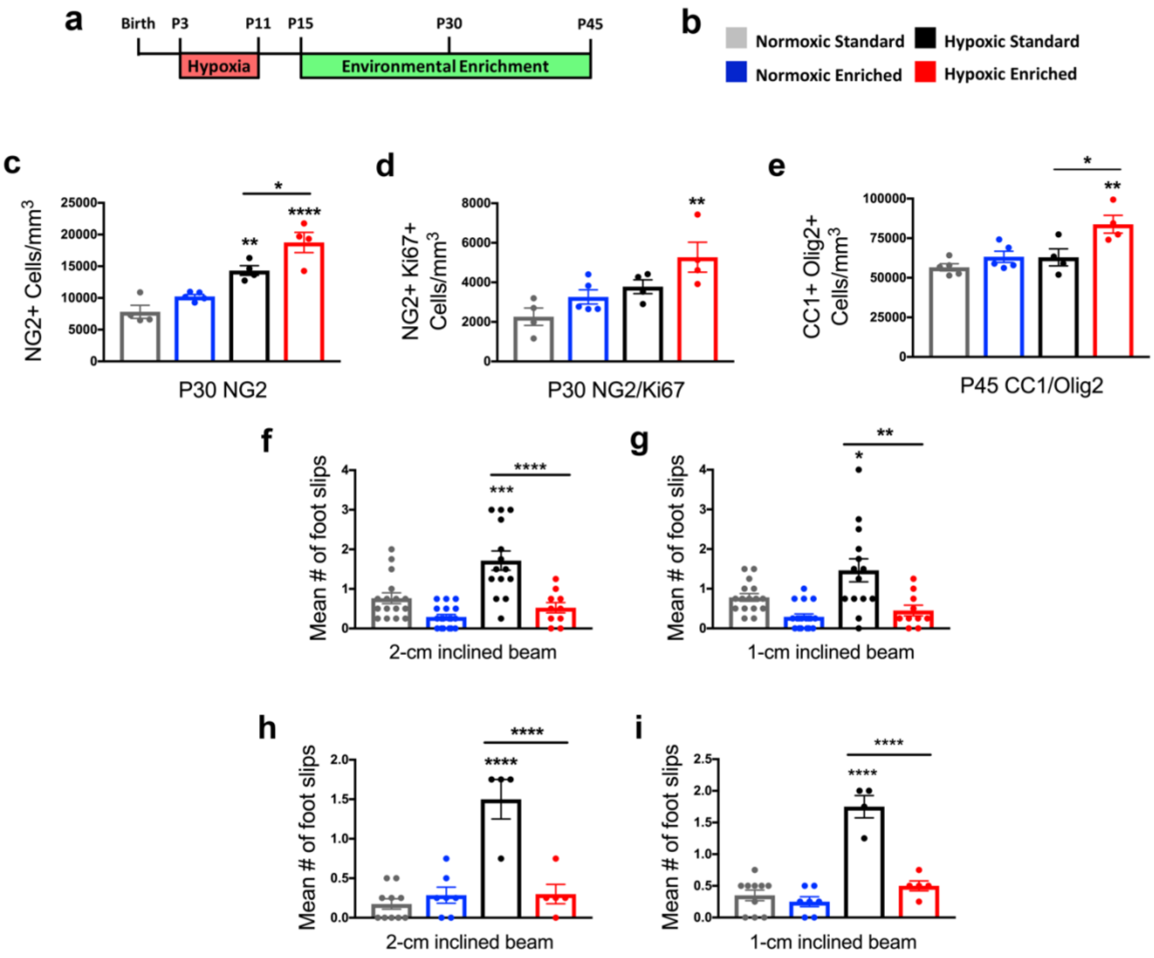


**Supplementary Figure 11. Replication of major findings in CNP-bacTRAP and PDGFRα-bacTRAP mice. (a)** Experimental paradigm. *CNP-bacTRAP* and *PDGFRα-bacTRAP* mice were exposed to hypoxic conditions (HX) from postnatal day 3 (P3) to P11, followed by environmental enrichment (EE) from P15 to P45. Mice were sacrificed for analysis at P30 and P45. **(b)** Experimental groups. **(c)** Quantification of NG2+ OPCs at P30 in *CNP-bacTRAP* mice (* $p = 0.04$, ** $p = 0.003$, **** $p < 0.0001$) (NX, $n = 4$; NX-EE, $n = 5$; HX, $n = 4$; HX-EE, $n = 4$). **(d)** Quantification of NG2+Ki67+ proliferating OPCs at P30 in *CNP-bacTRAP* mice (** $p = 0.005$) (NX, $n = 4$; NX-EE, $n = 5$; HX, $n = 4$; HX-EE, $n = 4$). **(e)** Quantification of CC1+OLIG2+ OLs at P45 in *CNP-bacTRAP* mice (* $p = 0.02$, ** $p = 0.002$) (NX, $n = 5$; NX-EE, $n = 5$; HX, $n = 4$; HX-EE, $n = 4$). **(f-g)** Quantification of foot slips in *CNP-bacTRAP* mice for 2-cm beam (*** $p = 0.0002$, **** $p < 0.0001$) **(f)**, and 1-cm beam (* $p = 0.02$, ** $p = 0.001$) **(g)** at P45 (NX, $n = 16$; NX-EE, $n = 18$; HX, $n = 14$; HX-EE, $n = 10$). **(h-i)** Quantification of foot slips in *PDGFRα-bacTRAP* for 2-cm beam **(h)**, and 1-cm beam **(i)** at P45 (**** $p < 0.0001$) (NX, $n = 10$; NX-EE, $n = 7$; HX, $n = 4$; HX-EE, $n = 5$). Data expressed as mean ± SEM. One-way ANOVA followed by a Tukey's post hoc test for

multiple comparisons was used to compare differences between groups. Source data are provided as a Source Data file.
