# Supplementary material for: Environmental enrichment ameliorates perinatal brain injury and promotes functional white matter recovery
Source: Nat Commun. 2020 Feb 19;11:964. doi: 10.1038/s41467-020-14762-7 (PMC7031237; doi:10.1038/s41467-020-14762-7)
Supplement: Supplementary file 3 — Reporting Summary [file 41467_2020_14762_MOESM3_ESM.pdf]

nature research

Corresponding author(s): Vittorio Gallo

Last updated by author(s): Jan 28, 2020

Reporting Summary

Nature Research wishes to improve the reproducibility of the work that we publish. This form provides structure for consistency and transparency in reporting. For further information on Nature Research policies, see Authors & Referees and the Editorial Policy Checklist .

Statistics

For all statistical analyses, confirm that the following items are present in the figure legend, table legend, main text, or Methods section.

n/a	Confirmed	
☐	☒	The exact sample size (*n*) for each experimental group/condition, given as a discrete number and unit of measurement
☐	☒	A statement on whether measurements were taken from distinct samples or whether the same sample was measured repeatedly
☐	☒	The statistical test(s) used AND whether they are one- or two-sided<br>*Only common tests should be described solely by name; describe more complex techniques in the Methods section.*
☒	☐	A description of all covariates tested
☐	☒	A description of any assumptions or corrections, such as tests of normality and adjustment for multiple comparisons
☐	☒	A full description of the statistical parameters including central tendency (e.g. means) or other basic estimates (e.g. regression coefficient) AND variation (e.g. standard deviation) or associated estimates of uncertainty (e.g. confidence intervals)
☐	☒	For null hypothesis testing, the test statistic (e.g. *F*, *t*, *r*) with confidence intervals, effect sizes, degrees of freedom and *P* value noted<br>*Give P values as exact values whenever suitable.*
☒	☐	For Bayesian analysis, information on the choice of priors and Markov chain Monte Carlo settings
☒	☐	For hierarchical and complex designs, identification of the appropriate level for tests and full reporting of outcomes
☒	☐	Estimates of effect sizes (e.g. Cohen's *d*, Pearson's *r*), indicating how they were calculated

Our web collection on statistics for biologists contains articles on many of the points above.

Software and code

Policy information about availability of computer code

Data collection	Immunohistochemical data was collected using Zeiss LSM or Zen 2.3 software. RNA-sequencing was performed on Novaseq 6000 (Illumina).
Data analysis	Images were viewed and analyzed using imageJ software (version 2.0.0, NIH). Data was analyzed using Microsoft Excel (version 16.16.4), Graphpad/Prism (version 7), Ingenuity Pathway Analysis (version 1-16, Qiagen), STAR (version 2.5 and 2.6), HTseq (version 0.10.0), and DESeq2 (version 1.20).

For manuscripts utilizing custom algorithms or software that are central to the research but not yet described in published literature, software must be made available to editors/reviewers. We strongly encourage code deposition in a community repository (e.g. GitHub). See the Nature Research guidelines for submitting code & software for further information.

Data

Policy information about availability of data

All manuscripts must include a data availability statement. This statement should provide the following information, where applicable:

- Accession codes, unique identifiers, or web links for publicly available datasets
- A list of figures that have associated raw data
- A description of any restrictions on data availability

All RNA-sequencing data has been uploaded to the Sequence Read Archive (NCBI). Accession code: PRJNA597018. All other data is available in the source file.

Field-specific reporting

Please select the one below that is the best fit for your research. If you are not sure, read the appropriate sections before making your selection.

- [x] Life sciences
- [ ] Behavioural & social sciences
- [ ] Ecological, evolutionary & environmental sciences

For a reference copy of the document with all sections, see nature.com/documents/nr-reporting-summary-flat.pdf

Life sciences study design

All studies must disclose on these points even when the disclosure is negative.

Sample size	This sample sizes for these experiments were based on similar experiments done in the lab using the same injury model (Jablonska et al., 2012; Scafidi et al., 2014). The sample size of all experiments in this study was a minimum of 3 animals, as is standard in the field. Behavioral experiments required larger sample sizes (~8-15 animals).
Data exclusions	No data were excluded from this study.
Replication	All experiments were replicated on numerous animals, collected across many days. The primary cellular and behavioral findings were successfully replicated in multiple mouse strains (CNP-EGFP and bacTRAP mice) by individual researchers over long periods of time.
Randomization	Mice were randomly assigned to experimental groups.
Blinding	Experimenters were blinded to experimental group for all analyses except for RNA-sequencing experiments, when experimenters needed to be unblinded in order to perform the proper comparisons.

Reporting for specific materials, systems and methods

We require information from authors about some types of materials, experimental systems and methods used in many studies. Here, indicate whether each material, system or method listed is relevant to your study. If you are not sure if a list item applies to your research, read the appropriate section before selecting a response.

Materials & experimental systems

n/a	Involved in the study	
[ ]	[x]	Antibodies
[x]	[ ]	Eukaryotic cell lines
[x]	[ ]	Palaeontology
[ ]	[x]	Animals and other organisms
[x]	[ ]	Human research participants
[x]	[ ]	Clinical data

Methods

n/a	Involved in the study	
[x]	[ ]	ChIP-seq
[x]	[ ]	Flow cytometry
[x]	[ ]	MRI-based neuroimaging

Antibodies

Antibodies used	Primary: Rat anti-BrdU (ab6326; Abcam), rabbit anti-GFAP (ab7260; Abcam), mouse anti-SMI31 (801601; Biolegend), mouse anti-SMI32 (801701; Biolegend), rabbit anti-IBA1 (019-19741; Wako), rabbit anti-CD68 (ab125212; Abcam), rabbit anti-NG2 (AB5320; Millipore), rabbit anti-Olig2 (AB9610; Millipore), rabbit anti-Ki67 (ab16667; Abcam), and mouse anti-CC1 (OP80; Calbiochem), rat anti-PDGFRα (553731; BD Biosciecnces), rabbit anti-cleaved caspase 3 (9664; Cell Signaling), mouse anti-MBP (SMI-99P; Covance), mouse anti-Actin (MAB1501R; Millipore), and mouse anti-MAG (sc-15324; Santa Cruz Biotechnology). Secondary: Alexa Fluor 647 - donkey anti-mouse (715-605-150; Jackson Immunoresearch), Alexa Fluor 594 - donkey anti-rabbit (711-585-152; Jackson Immunoresearch), Alexa Fluor 647 donkey anti-rat (712-605-150; Jackson Immunoresearch), HRP-conjugated goat anti-rabbit (sc-2004; Santa Cruz), and HRP-conjugated goat anti-mouse (sc-2005; Santa Cruz).
Validation	All primary antibodies have either been validated for immunohistochemistry or western blots for use on mouse. More information, including citations, can be found on manufactures websites.

Animals and other organisms

Policy information about studies involving animals; ARRIVE guidelines recommended for reporting animal research

Laboratory animals	Male and female mice ranging from birth through postnatal day 45 were used for this study. Strains used include: CNP-EGFP (generated by Dr. V. Gallo, Children's National Health System, Washington, DC), C57BL/6 (The Jackson Laboratory #003548), CD1 (Crl:CD1(ICR)); Charles River), B6: 129-MyRFtm1Barr/J (The Jackson Laboratory #010607), PDGFRα-CreERT2 (The Jackson Laboratory #018280), MyRF(flox/flox) (The Jackson Laboratory #010607), Rosa26-YFP (The Jackson Laboratory #006148), CNP-bacTRAP (The Jackson Laboratory #009159) and PDGFRα-bacTRAP (The Jackson Laboratory #030268)

Wild animals	No wild animals were used in this study.
Field-collected samples	No field-collected samples were used in this study.
Ethics oversight	All animal procedures were performed according to the Institutional Animal Care and Use Committee (IACUC) of the Children's National Health System (protocol #30473) and the Guide for the Care and Use of Laboratory Animals (National Institutes of Health).

Note that full information on the approval of the study protocol must also be provided in the manuscript.
