# Supplementary material for: Environmental enrichment ameliorates perinatal brain injury and promotes functional white matter recovery
Source: Nat Commun. 2020 Feb 19;11:964. doi: 10.1038/s41467-020-14762-7 (PMC7031237; doi:10.1038/s41467-020-14762-7)
Supplement: Supplementary file 4 — Description of Additional Supplementary Files [file 41467_2020_14762_MOESM4_ESM.docx]

**Description of Additional Supplementary Files**

File name: Supplementary Data 1
Description: All DEGs for PDGF-TRAP P18 HX vs NX comparison, ranked by fold-change. The “Gene” column lists entrez gene names. “baseMean” refers to the mean normalized counts for all samples. ”padj” refers to the p-value that was determined using the Wald test with Benjamini-Hochberg post hoc.

File name: Supplementary Data 2
Description: All DEGs for PDGF-TRAP P22 HX vs NX comparison, ranked by fold-change. The “Gene” column lists entrez gene names. “baseMean” refers to the mean normalized counts for all samples. ”padj” refers to the p-value that was determined using the Wald test with Benjamini-Hochberg post hoc.

File name: Supplementary Data 3
Description: All DEGs for PDGF-TRAP P30 HX vs NX comparison, ranked by fold-change. The “Gene” column lists entrez gene names. “baseMean” refers to the mean normalized counts for all samples. ”padj” refers to the p-value that was determined using the Wald test with Benjamini-Hochberg post hoc.

File name: Supplementary Data 4
Description: All DEGs for PDGF-TRAP P18 HX-EE vs HX comparison, ranked by fold-change. The “Gene” column lists entrez gene names. “baseMean” refers to the mean normalized counts for all samples. ”padj” refers to the p-value that was determined using the Wald test with Benjamini-Hochberg post hoc.

File name: Supplementary Data 5
Description: All DEGs for PDGF-TRAP P22 HX-EE vs HX comparison, ranked by fold-change. The “Gene” column lists entrez gene names. “baseMean” refers to the mean normalized counts for all samples. ”padj” refers to the p-value that was determined using the Wald test with Benjamini-Hochberg post hoc.

File name: Supplementary Data 6
Description: All DEGs for PDGF-TRAP P30 HX-EE vs HX comparison, ranked by fold-change. The “Gene” column lists entrez gene names. “baseMean” refers to the mean normalized counts for all samples. ”padj” refers to the p-value that was determined using the Wald test with Benjamini-Hochberg post hoc.

File name: Supplementary Data 7
Description: All DEGs for CNP-TRAP P22 HX vs NX comparison, ranked by fold-change. The “Gene” column lists entrez gene names. “baseMean” refers to the mean normalized counts for all samples. ”padj” refers to the p-value that was determined using the Wald test with Benjamini-Hochberg post hoc.

File name: Supplementary Data 8
Description: All DEGs for CNP-TRAP P30 HX vs NX comparison, ranked by fold-change. The “Gene” column lists entrez gene names. “baseMean” refers to the mean normalized counts for all samples. ”padj” refers to the p-value that was determined using the Wald test with Benjamini-Hochberg post hoc.

File name: Supplementary Data 9
Description: All DEGs for CNP-TRAP P45 HX vs NX comparison, ranked by fold-change. The “Gene” column lists entrez gene names. “baseMean” refers to the mean normalized counts for all samples. ”padj” refers to the p-value that was determined using the Wald test with Benjamini-Hochberg post hoc.

File name: Supplementary Data 10
Description: All DEGs for CNP-TRAP P22 HX-EE vs HX comparison, ranked by fold-change. The “Gene” column lists entrez gene names. “baseMean” refers to the mean normalized counts for all samples. ”padj” refers to the p-value that was determined using the Wald test with Benjamini-Hochberg post hoc.

File name: Supplementary Data 11
Description: All DEGs for CNP-TRAP P30 HX-EE vs HX comparison, ranked by fold-change. The “Gene” column lists entrez gene names. “baseMean” refers to the mean normalized counts for all samples. ”padj” refers to the p-value that was determined using the Wald test with Benjamini-Hochberg post hoc.

File name: Supplementary Data 12
Description: All DEGs for CNP-TRAP P45 HX-EE vs HX comparison, ranked by fold-change. The “Gene” column lists entrez gene names. “baseMean” refers to the mean normalized counts for all samples. ”padj” refers to the p-value that was determined using the Wald test with Benjamini-Hochberg post hoc.
