# Supplementary material for: Environmental enrichment ameliorates perinatal brain injury and promotes functional white matter recovery
Source: Nat Commun. 2020 Feb 19;11:964. doi: 10.1038/s41467-020-14762-7 (PMC7031237; doi:10.1038/s41467-020-14762-7)
Supplement: Supplementary file 2 — Peer Review [file 41467_2020_14762_MOESM2_ESM.pdf]

Reviewers' comments:

Reviewer #1 (Remarks to the Author):

Summary:

In this study, the authors show that sustained environmental enrichment can increase OPC proliferation, oligodendrocyte density, the fraction of myelinated axons and myelin thickness to improve behavioural outcomes following developmental hypoxia. Restricting the period of environmental enrichment was found to diminish or negate its benefit, which was directly reliant on new oligodendrocyte production by OPCs. The authors also show that male and female mice respond equivalently to environmental enrichment, but that physical activity, social enrichment or novelty alone are insufficient to overcome the pathological and behavioural consequences of developmental hypoxia.

Overall this appears to be a very thorough and appropriately analysed study.

Major comments:

This manuscript contains a large quantity of data that will be of interest to researchers in the myelin, neural plasticity and behavioural plasticity fields. But the overall conclusion is that environmental enrichment enhances myelination following hypoxia. It is unfortunate that the RNA sequencing experiment stops short of explaining: (i) how hypoxia renders the brain responsive to environmental manipulation, or (ii) how the environmental enrichment has its effect. The authors compare oligodendrocyte RNA taken from the subcortical white matter of hypoxia and hypoxia + enrichment mice. They show that environmental enrichment alters gene expression by oligodendrocytes, upregulating genes involved in metabolic activity including lipid metabolism, cellular morphology (cytoskeletal dynamics), and oligodendrocyte maturation / myelination. These changes in gene expression may simply reflect RNA isolated from an oligodendrocyte population that contains more oligodendrocytes that are actively maturing and myelinating. It is not clear that this experiment provides mechanistic insight into how the enrichment is working.

Minor comments:

1) The experimental manipulations and comparisons used in this study are standard for the field, yet the manuscript is unnecessarily complex in places. I suggest the authors go over the paper and try to find a more direct way to explain their study. To give two examples of sentences they may like to reword: “In spite of many challenges, the intersection of WM development and perinatal brain injury is a maturational period primed for plasticity and functional improvement induced by exogenous environmental stimuli.” And “Nevertheless, our understanding and clinical implementation of environmental influence on developmental myelination, especially after injury, is nascent.”

2) From the methods, the age of weaning is not clear. Please indicate whether the cages are individually ventilated. How many mice are co-housed in the standard housing? In the TEM and behavioural sections, the authors indicate that quantification was performed blind to experimental conditions. Was that also true for the other histological analyses? What test was performed to assess normality prior to applying the chosen parametric or non-parametric statistical tests?

3) It would be beneficial if the authors included a schematic in an early figure or one of the supplementary figures clearly showing the area they are defining and analysing as subcortical white matter. It would also be helpful to have some example histological panels showing expression of the oligodendroglial markers at P30 and P45, under normoxic and hypoxic conditions +/- enrichment, at least for the sustained enrichment condition.

4) At the timepoint analysed in Figure 1 (P45), the density of CC1+ oligodendrocytes is elevated with hypoxia, and environmental enrichment produces a further (small) increase in oligodendrocyte density. Can the authors please clarify whether developmental hypoxia initially causes a reduction in the number of CC1+ oligodendrocytes (relative to normoxia) prior to this increase in oligodendrocyte number?

5) The authors hypothesise that hypoxia reopens a critical window of developmental myelination required for the beneficial effects of EE. Are they implying that in normal development there is an earlier window where EE would have an effect under normoxic conditions?

6) In Figure 2, it is interesting that only hypoxia + enrichment restores the fraction of myelinated axons to normoxic levels, while mice experiencing hypoxia alone have fewer myelinated axons. Please explain this observation in light of hypoxia and hypoxia + enrichment having an elevated number of CC1+ cells at P45 and both have elevated MAG and MBP expression compared to normoxic controls. (i) Are the elevated number of CC1+ cells detected under hypoxic conditions premyeliniating cells? You could look at this by staining to detect BCAS1. (ii) How do hypoxic mice have fewer myelinated axons than all other conditions by have elevated MAG and MBP? From the g-ratio data, their myelin does not appear to be abnormally thick. What is this likely to reflect?

7) You use the terms oligodendrocyte progenitor cells (OPCs), OL progenitors and proliferating OLs on page 5 of the manuscript. These terms are somewhat ambiguous, as they could all mean the same thing. From Figure 1, it appears the authors are quantifying the density of OPCs (CNS-EGFP+ NG2+; Fig.1 C), separately from the density of oligodendrocytes (CNP-EGFP+ CC1+; Fig. 1E). They also quantify the density of cells within the oligodendrocyte lineage that are actively dividing (CNP-EGFP+ Ki67+; Fig. 1D). This is an unusual measure but effectively equates to the density of proliferating OPCs. I have no issue with these data but ask the authors to define this measurement in text, explaining clearly what it best equates to, before using a single term to describe it.

8) Similarly, on page 5, please be cautious with your use of the term "mature OLs" when you are only quantifying CC1 expression. You are counting oligodendrocytes, not mature OLs, as Figure 1 does not provide any indication as to the level of maturity i.e. myelinating OLs. Please refer to them as OLs not mature OLs.

9) Running has no effect on OPC or OL density, and only a very small effect on OLIG2+ cell density. Therefore, it is unclear why the authors suggest that running "primes" the system so that environmental enrichment can impact oligodendrogenesis and maturation after hypoxia.

10) The graph in Figure 6G of hypoxia v hypoxia + enrichment is the same as Figure 2F. It is not necessary to reshow the same data since no new statistical comparison is being made. Please just refer back to the original comparison in Fig. 2F.

11) The presentation of Figure 6B-D as line graphs makes the statistical comparisons more difficult to follow. For example, do the asterisks in Fig. 6B over P30 relate to early enrichment being different to sustained enrichment? The cellular response induced by hypoxia + early enrichment seems a transient increase in OPC density, that is the same as hypoxia standard housing by P45; early enrichment produces no change in the density of proliferating OPCs relative to hypoxia + standard housing; and oligodendrocyte density appears to be less then hypoxia standard housing prior to P45. Please present these data as a time series of bar graphs, so the comparisons can be more easily seen. This effect also doesn't seem to warrant the 2 ++ rating of a cellular effect in Supplementary Figure 6 - which is why I am concerned the line graph prevents a full appreciation of these data.

Reviewer #2 (Remarks to the Author):

This study by Forbes et al. focuses on the impact of environmental enrichment following perinatal brain injury. They provide important new data indicating that environmental enrichment improves recovery both at the cellular level and the behavioral level following perinatal hypoxia, a model that they have studied extensively. The data provided here are novel and important for our understanding of potential therapeutic approaches to perinatal hypoxia in children. The authors demonstrate that following perinatal hypoxic, there is significant proliferation and accumulation of oligodendrocyte progenitor cells and maturing cells, and that this is enhanced when the animals are in an enriched environment. The most striking data about the oligodendrocyte responses comes from the ultrastructure, where very few myelinated axons are found at P45 after the hypoxic period, whereas there was a significant increase in the number of myelinated fibers for these animals when maintained in the enriched environment. Importantly, when the ability of oligodendrocytes to differentiate to myelinating cells was blocked, the recovery outcomes were blocked. Thus the production of newly myelinating cells was crucial for the enhanced cellular and behavioral outcomes. The RNA-seq data in the latter figures is clearly of interest and having the tables available will be important for other investigators, but in general the RNAs identified in the hypoxia-enriched environment group of animals are consistent with what would be expected for cells that are differentiating more to myelinating cells and generating myelin. Nevertheless, some intriguing RNA data may come from this analysis and it is very useful to have as a novel database of oligodendrocyte gene expression. Overall, these data all provide strong support that the enriched environment model investigated here is valuable for studying how the brain can respond to hypoxic injury and they clearly support an essential role for oligodendrocytes and new myelination in recovery from perinatal hypoxic damage.
Some points to consider:
They quantified cells from corpus callosum, external capsule and cingulum. It appears that the numbers in the graphs are a combination of the numbers from individual regions. That is indicated in the methods, which are supplemental. This would be faster for the reader to understand if included in the figure legends as well, or at least the figure 1 legend. Additionally, a supplemental figure showing the data from the different regions would be useful.
As they note that there are white matter volume changes after hypoxia, and they are quantifying the density of cells, does the total number of cells change if the entire volume of white matter is considered?
There is a clear focus on oligodendrocyte responses, which are essential for the positive outcomes. Nevertheless, the behavior changes result from overall tissue responses. It would be useful to assess whether other cells also increase proliferation during or after the hypoxia period or during enrichment. Are there increased microglia or astrocytes in white matter domains under investigation? Are there significant axonal changes in this tissue?
Points of clarification:
1. The animals raised in the enriched environments were maintained in larger cages. It should be stated that the control animals were also in larger cages with larger numbers of animals, assuming that to be the case.
2. No discussion of Figure 6A-D is presented, which focused on the cells in the early intervention context, and there is discussion of supplemental figure 3, which is also focused on the cells from the early intervention context. Perhaps these data could be discussed together?
3. It would be good to define the colors in Supplemental Figure 7. They are consistent with earlier graphs, so can be guessed, but should be defined here as well.

Reviewer #3 (Remarks to the Author):

The study, by Forbes and colleagues, demonstrates, using an impressive range of complementary methods, that prolonged complete environment enrichment (EE: incl. social, novel object and exercise) increases myelination and ameliorates beam walking after perinatal hypoxia, but short or delayed EE does not. The authors find that EE increases OPC proliferation and differentiation, as well

as myelination. They then show, by knocking out the myelin regulatory transcription factor (myrf) in OPCs (thus preventing OPC from differentiating into myelinating oligodendrocytes), that amelioration of EE on beam walking depends on OPC differentiation, or oligodendrogenesis. The authors further investigate the molecular pathways involved by using a novel mouse model in which ribosomes in oligodendrocytes have been tagged with GFP, allowing the authors to specifically identify mRNAs that are being actively transcribed in myelinating oligodendrocytes. The findings are potentially very interesting, and of particular interest is the fact that the effect of EE is apparently time sensitive, suggesting that there is a window of opportunity for regeneration after hypoxia. Nevertheless, questions regarding the interpretation of the data remain, and further evidence is needed to support some of the claims made in the manuscript.

Major comments
(1) The authors claim EE is selective in promoting proliferation and differentiation, as well as myelination, after hypoxia. However, it seems that EE is in fact enhancing an existing response, there is similar fold augmentation between normoxia vs hypoxia and hypoxia vs EE. This enhancement and the outcome with greater myelination could perhaps be better explained by EE increasing OLs survival, particularly as it has previously been shown that active neurons promote OLs survival (Barres & Raff Nature 1993), and that during development there is an overproduction of OLs, of which around half die (Barres et al., Cell 1992). Therefore, it is likely that rather than EE selectivity promoting more OL proliferation and differentiation, it is promoting cell survival, particularly as most differentially-expressed genes the authors find between hypoxia and EE-hypoxia are cell survival genes. The authors should control for this possibility.

(2) The window of opportunity to augment behavioral outcomes after prenatal hypoxia is an interesting and important notion. However, it is not clear to this reviewer which is more important: (i) the time after hypoxia, e.g. when animals receive EE exposure; or (ii) the duration the animals are exposed to EE. As with all variables of the 'normal' post hypoxia EE exposure, the duration was 30 days' exposure vs 10 days' exposure (at different timepoints after hypoxia). Therefore, the question of whether 30 days' duration at a later timepoint than P15 would have the same effect remains. This is important for establishing whether there is a window of opportunity or not.

(3) The authors state in the abstract that they "identify molecular pathways involved in the enrichment-induced oligodendrocyte response to hypoxic brain injury." The molecular pathways 'identified' seem to be mainly related to cell survival and myelination. This is not surprising, given that with hypoxia EE animals myelinate, whereas with hypoxia alone they do not (Figure 2). The information about the molecular pathways involved is not provided clearly. More work is needed to determine the molecular pathways that differentiate between the two and can provide explanation as to why EE promotes myelination and/or why in hypoxia alone OLs fail to myelinate despite robust increases in cell numbers. Perhaps it would have been more informative to focus on the OPCs.

Minor comments
(i) Why is the cellular response and outcome of EE different between TRAP mice and the CNP-EGFP mice (see Figure 1 vs Supplementary Figure 9). Moreover, are the EE-HX TRAP mice showing better outcomes than the normoxia control on the inclined beam test (Figure 9 F,G)?

(ii). In Figure 4D,C,E, is the difference between normoxia and normoxia EE significant? It seems that normoxia EE has fewer foot slips and is quicker than normoxia. What is the p value? Could it be that there is an increase in myelination with EE exposure? This is perhaps why there is such a great variance in Figure 2D,E (it might be better to quantify the proportion of myelinated axons per unmyelinated axons rather than the field of view).

(iii) Representative images for barographs would be useful for interpreting the data in the manuscript.

(iv) Figure 2 is missing information regarding which white matter tract is being quantified and where is it being quantified. Moreover, why is there such a great variance in the normox EE group and what is the cause of that? Are exactly the same locations within the white matter tact being compared?

(v) On page 5, line 181, define “smaller.” Quantify the difference and indicate whether it is significant or not. More generally, when comparing data throughout the manuscript, please give the average numbers and s.e.m., or the fold increase/decrease and/or p-value.

(vi) The resolution of the axis labels on bar graphs is poor. Labels and numbers are illegible. Please adjust.

(vii) The referencing is not consistent. It needs to be made uniform.

We are grateful for the reviewers' careful assessment of our work, and constructive comments aimed at improving the manuscript. Thank you for the opportunity to address these comments and submit a revised version of our work. We have now completed many new experiments and added substantial new data that strengthen our conclusions and greatly improve the manuscript. Below, we address all reviewers' comments in a point-by-point manner. For clarity, we have highlighted all major changes to the manuscript in grey.

Reviewer #1 (Remarks to the Author):

Summary:

In this study, the authors show that sustained environmental enrichment can increase OPC proliferation, oligodendrocyte density, the fraction of myelinated axons and myelin thickness to improve behavioural outcomes following developmental hypoxia. Restricting the period of environmental enrichment was found to diminish or negate its benefit, which was directly reliant on new oligodendrocyte production by OPCs. The authors also show that male and female mice respond equivalently to environmental enrichment, but that physical activity, social enrichment or novelty alone are insufficient to overcome the pathological and behavioural consequences of developmental hypoxia.

Overall this appears to be a very thorough and appropriately analysed study.

Major comments:

This manuscript contains a large quantity of data that will be of interest to researchers in the myelin, neural plasticity and behavioural plasticity fields. But the overall conclusion is that environmental enrichment enhances myelination following hypoxia. It is unfortunate that the RNA sequencing experiment stops short of explaining: (i) how hypoxia renders the brain responsive to environmental manipulation, or (ii) how the environmental enrichment has its effect. The authors compare oligodendrocyte RNA taken from the subcortical white matter of hypoxia and hypoxia + enrichment mice. They show that environmental enrichment alters gene expression by oligodendrocytes, upregulating genes involved in metabolic activity including lipid metabolism, cellular morphology (cytoskeletal dynamics), and oligodendrocyte maturation / myelination. These changes in gene expression may simply reflect RNA isolated from an oligodendrocyte population that contains more oligodendrocytes that are actively maturing and myelinating. It is not clear that this experiment provides mechanistic insight into how the enrichment is working.

- *We appreciate the thoughtful analysis from Reviewer #1, and believe we have added substantial data that will help to resolve the major concerns raised. To address how hypoxia renders the brain responsive to environmental manipulation, we have now included complete time-course analyses of HX-induced OL lineage-specific changes in the translatome. To do this, we added RNA-sequencing results from normoxic standard*

control mice and compared them to hypoxic standard mice at various time points following HX. These data reveal multiple interesting findings about endogenous OL-lineage cell responses to HX. One finding is that – at certain time points – HX induces changes in OPCs and OLs that reveal more active cell maturation processes, likely indicative of a regenerative response in the OL-lineage. For example, at P18 OPCs have numerous cellular functions predicted to be inactivated, as compared to normoxia, but at P22 and P30 many of these same functions appear to be more active in OPCs after HX. At P22, predicted functions related to cell maturation also appear to be more active in HX OLs. This likely supports Reviewer #1's suggestion that gene expression changes are reflective of RNA isolated from OL populations with different maturational statuses. However, at P30, HX induces inactivation of a variety of predicted cell functions in OLs that are closely associated with myelination. Furthermore, differences in the translatomes of hypoxic and normoxic OLs are virtually nonexistent by P45, despite persistent myelin deficits (EM data). Lastly, HX causes an accumulation of premyelinating OLs (ENPP6-exressing cells), that is partially reversed with EE. These data suggest that HX causes OL-specific impairment, and that there is a rather short window of opportunity for timely environmental intervention to effectively reverse or attenuate the effects of HX.

Because EE is a global approach to treating WM injury following HX, we believe mechanisms of action to be multifaceted and complex, involving multiple cell types. This notion is not only supported by our translatome analysis, but also by all our cellular studies demonstrating EE effects on OPCs and OLs at multiple levels. As alluded to above, we have now also added substantial new sequencing data for OPCs (using PDGFRα-bacTRAP mice), to provide a more complete perspective of OL-lineage changes after HX, with and without enrichment. Additionally, we have performed immunohistochemical analyses for other cell types with potential involvement (as requested by Reviewer #2). We determined that astrocytes and microglia are likely not playing major roles in the EE-induced WM recovery after HX. However, abnormal neurofilament phosphorylation in WM axons that is partially reversed with EE, indicates that pyramidal neurons and their commissural axons are undoubtedly involved in the recovery from HX. In sum, we believe that the substantial new data added to the revised version of this manuscript addresses Reviewer #1's major concerns, and provides a more complete perspective of how EE renders its effects.

Minor comments:

1) The experimental manipulations and comparisons used in this study are standard for the field, yet the manuscript is unnecessarily complex in places. I suggest the authors go over the paper and try to find a more direct way to explain their study. To give two examples of sentences they may like to reword: "In spite of many challenges, the intersection of WM development and perinatal brain injury is a maturational period primed for plasticity and functional improvement induced by exogenous environmental stimuli." And "Nevertheless, our

understanding and clinical implementation of environmental influence on developmental myelination, especially after injury, is nascent."

- *The manuscript was substantially edited to provide clear and direct explanations of the experiments performed.*

2) From the methods, the age of weaning is not clear. Please indicate whether the cages are individually ventilated. How many mice are co-housed in the standard housing? In the TEM and behavioural sections, the authors indicate that quantification was performed blind to experimental conditions. Was that also true for the other histological analyses? What test was performed to assess normality prior to applying the chosen parametric or non-parametric statistical tests?

- *In order to provide a more complete description of our procedures, we amended the methods section to include all of the items raised by Reviewer #1. In the "Animals" section, we provided age of weaning information (P21), and included that every cage used in these experiments were individually ventilated. In the "Environmental enrichment" section, we added a more detailed description of the standard environment, which consists of between 3-5 mice per cage. In the "Image acquisition and analysis" section, we stated that all histological analyses were performed in a blinded manner. Lastly, in the "Statistical analysis" section, we added that D'Agostino & Pearson normality tests were performed to confirm that our data were normally distributed.*

3) It would be beneficial if the authors included a schematic in an early figure or one of the supplementary figures clearly showing the area they are defining and analysing as subcortical white matter. It would also be helpful to have some example histological panels showing expression of the oligodendroglial markers at P30 and P45, under normoxic and hypoxic conditions +/- enrichment, at least for the sustained enrichment condition.

- *A schematic representation of coronal section with corresponding areas of analysis was added to Figure 1.*
- *Representative images for all oligodendroglial markers was added to Supplemental Figure 1.*

4) At the timepoint analysed in Figure 1 (P45), the density of CC1+ oligodendrocytes is elevated with hypoxia, and environmental enrichment produces a further (small) increase in oligodendrocyte density. Can the authors please clarify whether developmental hypoxia initially causes a reduction in the number of CC1+ oligodendrocytes (relative to normoxia) prior to this increase in oligodendrocyte number?

- *Yes, hypoxia causes an initial loss of CC1+ oligodendrocytes that our group has previously published. The following sentence, with citations, was added to the manuscript for clarity: "This aligns with previous studies that reported an initial loss of*

OLs in the WM recovers approximately one month following HX injury (Jablonska et al., 2012; Scafidi et al., 2014)." Page 6, first paragraph.

5) The authors hypothesise that hypoxia reopens a critical window of developmental myelination required for the beneficial effects of EE. Are they implying that in normal development there is an earlier window where EE would have an effect under normoxic conditions?

- *We agree with reviewer #1 and believe that a critical window is opened, but not reopened. We have edited the manuscript reflect this change.*

6) In Figure 2, it is interesting that only hypoxia + enrichment restores the fraction of myelinated axons to normoxic levels, while mice experiencing hypoxia alone have fewer myelinated axons. Please explain this observation in light of hypoxia and hypoxia + enrichment having an elevated number of CC1+ cells at P45 and both have elevated MAG and MBP expression compared to normoxic controls. (i) Are the elevated number of CC1+ cells detected under hypoxic conditions premyeliniating cells? You could look at this by staining to detect BCAS1. (ii) How do hypoxic mice have fewer myelinated axons than all other conditions by have elevated MAG and MBP? From the g-ratio data, their myelin does not appear to be abnormally thick. What is this likely to reflect?

- *To explain this very interesting and important observation, we quantified premyelinating OLs by performing in situ hybridization for ENPP6 (Xiao et al., 2016). We found that after HX, there was a significant increase in premyelinating OLs that was reduced with EE (Fig. 1m-n). Because premyelinating OLs also express CC1, this increase explains the discrepancy between elevated CC1 cells and low myelination in the HX mice. Furthermore, these premyelinating OLs have previously been described as expressing myelin proteins MAG and MBP (Trapp et al., 1997). Thus, it would appear that these premyelinating OLs are producing myelin proteins (and possibly sheaths) despite not forming lasting myelin wraps.*

7) You use the terms oligodendrocyte progenitor cells (OPCs), OL progenitors and proliferating OLs on page 5 of the manuscript. These terms are somewhat ambiguous, as they could all mean the same thing. From Figure 1, it appears the authors are quantifying the density of OPCs (CNS-EGFP+ NG2+; Fig.1 C), separately from the density of oligodendrocytes (CNP-EGFP+ CC1+; Fig. 1E). They also quantify the density of cells within the oligodendrocyte lineage that are actively dividing (CNP-EGFP+ Ki67+; Fig. 1D). This is an unusual measure but effectively equates to the density of proliferating OPCs. I have no issue with these data but ask the authors to define this measurement in text, explaining clearly what it best equates to, before using a single term to describe it.

- *We have revised the manuscript to be more uniform when referring to different types of OL lineage cells. Additionally, we more clearly defined which markers equate to specific cell types.*

8) Similarly, on page 5, please be cautious with your use of the term “mature OLs” when you are only quantifying CC1 expression. You are counting oligodendrocytes, not mature OLs, as Figure 1 does not provide any indication as to the level of maturity i.e. myelinating OLs. Please refer to them as OLs not mature OLs.

- *We have revised the manuscript to replace “mature OLs” with “oligodendrocytes” or “differentiated OLs.”*

9) Running has no effect on OPC or OL density, and only a very small effect on OLIG2+ cell density. Therefore, it is unclear why the authors suggest that running “primes” the system so that environmental enrichment can impact oligodendrogenesis and maturation after hypoxia.

- *We agree with Reviewer #1 that there is insufficient evidence to suggest that running “primes” the system. We have removed this claim from the manuscript.*

10) The graph in Figure 6G of hypoxia v hypoxia + enrichment is the same as Figure 2F. It is not necessary to reshow the same data since no new statistical comparison is being made. Please just refer back to the original comparison in Fig. 2F.

- *We have removed the previously shown data from this figure (now figure 5).*

11) The presentation of Figure 6B-D as line graphs makes the statistical comparisons more difficult to follow. For example, do the asterisks in Fig. 6B over P30 relate to early enrichment being different to sustained enrichment? The cellular response induced by hypoxia + early enrichment seems a transient increase in OPC density, that is the same as hypoxia standard housing by P45; early enrichment produces no change in the density of proliferating OPCs relative to hypoxia + standard housing; and oligodendrocyte density appears to be less then hypoxia standard housing prior to P45. Please present these data as a time series of bar graphs, so the comparisons can be more easily seen. This effect also doesn’t seem to warrant the 2 ++ rating of a cellular effect in Supplementary Figure 6 - which is why I am concerned the line graph prevents a full appreciation of these data.

- *This figure (now figure 5) has been modified to reflect the reviewer’s request. Additionally, the 2++ rating in the summary figure (now figure 6) has been reduced to one “+”.*

Reviewer #2 (Remarks to the Author):

This study by Forbes et al. focuses on the impact of environmental enrichment following perinatal brain injury. They provide important new data indicating that environmental enrichment improves recovery both at the cellular level and the behavioral level following perinatal hypoxia, a model that they have studied extensively. The data provided here are novel and important for our understanding of potential therapeutic approaches to perinatal hypoxia in children. The authors demonstrate that following perinatal hypoxic, there is significant proliferation and accumulation of oligodendrocyte progenitor cells and maturing cells, and that this is enhanced when the animals are in an enriched environment. The most striking data about the oligodendrocyte responses comes from the ultrastructure, where very few myelinated axons are found at P45 after the hypoxic period, whereas there was a significant increase in the number of myelinated fibers for these animals when maintained in the enriched environment. Importantly, when the ability of oligodendrocytes to differentiate to myelinating cells was blocked, the recovery outcomes were blocked. Thus the production of newly myelinating cells was crucial for the enhanced cellular and behavioral outcomes. The RNA-seq data in the latter figures is clearly of interest and having the tables available will be important for other investigators, but in general the RNAs identified in the hypoxia-enriched environment group of animals are consistent with what would be expected for cells that are differentiating more to myelinating cells and generating myelin. Nevertheless, some intriguing RNA data may come from this analysis and it is very useful to have as a novel database of oligodendrocyte gene expression. Overall, these data all provide strong support that the enriched environment model investigated here is valuable for studying how the brain can respond to hypoxic injury and they clearly support an essential role for oligodendrocytes and new myelination in recovery from perinatal hypoxic damage.

Some points to consider:

They quantified cells from corpus callosum, external capsule and cingulum. It appears that the numbers in the graphs are a combination of the numbers from individual regions. That is indicated in the methods, which are supplemental. This would be faster for the reader to understand if included in the figure legends as well, or at least the figure 1 legend. Additionally, a supplemental figure showing the data from the different regions would be useful.

- *We have added a schematic representation to Figure 1, and we more clearly explain the regions quantified in the legend for Figure 1. Additionally, we have included WM region specific data Supplemental Figure 1 for the corresponding graphs (Figure 1d-f). These data show that no overt cell density differences exist between WM regions in our experiments.*

As they note that there are white matter volume changes after hypoxia, and they are quantifying the density of cells, does the total number of cells change if the entire volume of white matter is considered?

- *We analyzed and quantified cell densities in order to correct for any changes in white matter volume that might occur after hypoxia. Because our data is compiled from multiple sampled regions across the white matter, we predict that the changes we see in cell density are reflective of comparable changes in the total number of cells.*

There is a clear focus on oligodendrocyte responses, which are essential for the positive outcomes. Nevertheless, the behavior changes result from overall tissue responses. It would be useful to assess whether other cells also increase proliferation during or after the hypoxia period or during enrichment. Are there increased microglia or astrocytes in white matter domains under investigation? Are there significant axonal changes in this tissue?

- *To determine if other cell types were involved in behavioral changes, we performed immunohistochemical analyses of microglia, astrocytes and neurofilaments. There were no overt changes in microglia and astrocytes, however, hypoxia induced abnormal patterns of neurofilament phosphorylation that was reversed with EE. These data are included in supplementary figure 3.*

Points of clarification:
1. The animals raised in the enriched environments were maintained in larger cages. It should be stated that the control animals were also in larger cages with larger numbers of animals, assuming that to be the case.

- *Animals housed in standard conditions were not housed in larger cages. To help clarify this point, and to clearly state the number of mice housed in these conditions, a few sentences have been added to the "Environmental enrichment" methods section.*

2. No discussion of Figure 6A-D is presented, which focused on the cells in the early intervention context, and there is discussion of supplemental figure 3, which is also focused on the cells from the early intervention context. Perhaps these data could be discussed together?

- *These two sets of data are now discussed together in the last paragraph of page 10.*

3. It would be good to define the colors in Supplemental Figure 7. They are consistent with earlier graphs, so can be guessed, but should be defined here as well.

- *A legend defining groups with corresponding colors has been added to this figure (now Supp. 9).*

Reviewer #3 (Remarks to the Author):

The study, by Forbes and colleagues, demonstrates, using an impressive range of complementary methods, that prolonged complete environment enrichment (EE: incl. social, novel object and exercise) increases myelination and ameliorates beam walking after perinatal hypoxia, but short or delayed EE does not. The authors find that EE increases OPC proliferation and differentiation, as well as myelination. They then show, by knocking out the myelin regulatory transcription factor (myrf) in OPCs (thus preventing OPC from differentiating into myelinating oligodendrocytes), that amelioration of EE on beam walking depends on OPC differentiation, or oligodendrogenesis. The authors further investigate the molecular pathways involved by using a novel mouse model in which ribosomes in oligodendrocytes have been tagged with GFP, allowing the authors to specifically identify mRNAs that are being actively transcribed in myelinating oligodendrocytes. The findings are potentially very interesting, and of particular interest is the fact that the effect of EE is apparently time sensitive, suggesting that there is a window of opportunity for regeneration after hypoxia. Nevertheless, questions regarding the interpretation of the data remain, and further evidence is needed to support some of the claims made in the manuscript.

Major comments

(1) The authors claim EE is selective in promoting proliferation and differentiation, as well as myelination, after hypoxia. However, it seems that EE is in fact enhancing an existing response, there is similar fold augmentation between normoxia vs hypoxia and hypoxia vs EE. This enhancement and the outcome with greater myelination could perhaps be better explained by EE increasing OLs survival, particularly as it has previously been shown that active neurons promote OLs survival (Barres & Raff Nature 1993), and that during development there is an overproduction of OLs, of which around half die (Barres et al., Cell 1992). Therefore, it is likely that rather than EE selectivity promoting more OL proliferation and differentiation, it is promoting cell survival, particularly as most differentially-expressed genes the authors find between hypoxia and EE-hypoxia are cell survival genes. The authors should control for this possibility.

- *In order to test if differences in survival were present, we quantified cleaved caspase 3-expressing cells in the WM. At P22, P30, and P45, we found no significant differences in the number of apoptotic cells (OPCs or OLs), indicating apoptosis is not a contributing factor to the number of OL lineage cells at these time points following EE intervention. These data can be found in Supplemental Figure 2.*

(2) The window of opportunity to augment behavioral outcomes after prenatal hypoxia is an interesting and important notion. However, it is not clear to this reviewer which is more important: (i) the time after hypoxia, e.g. when animals receive EE exposure; or (ii) the duration the animals are exposed to EE. As with all variables of the 'normal' post hypoxia EE exposure, the duration was 30 days’ exposure vs 10 days’ exposure (at different timepoints after hypoxia). Therefore, the question of whether 30 days’ duration at a later timepoint than P15 would have the same effect remains. This is important for establishing whether there is a window of opportunity or not.

- *We thank the reviewer for raising this issue. This is a very important point that was overlooked for the initial submission of this manuscript. To address Reviewer #3's point, we performed an experiment in which mice were placed into EE from P25 to P55 - a full 30 days of EE, but 10 days delayed from the original paradigm. We discovered that this "delayed and extended" paradigm was insufficient to reproduce the EE-induced trends seen with our original paradigm (Supp. 6m-p), and also did not reverse the HX-induced behavioral deficit (Supp. 7j-l). This experiment highlights the importance of both timing and duration in the EE-induced recovery from HX.*

(3) The authors state in the abstract that they "identify molecular pathways involved in the enrichment-induced oligodendrocyte response to hypoxic brain injury." The molecular pathways 'identified' seem to be mainly related to cell survival and myelination. This is not surprising, given that with hypoxia EE animals myelinate, whereas with hypoxia alone they do not (Figure 2). The information about the molecular pathways involved is not provided clearly. More work is needed to determine the molecular pathways that differentiate between the two and can provide explanation as to why EE promotes myelination and/or why in hypoxia alone OLs fail to myelinate despite robust increases in cell numbers. Perhaps it would have been more informative to focus on the OPCs.

- *We appreciate Reviewer #3's concerns about the clarity of the RNA-sequencing data and our claim to have identified molecular pathways. We have now revised this language to reflect that we have only begun to identify molecular changes, as we predict that multiple mechanisms and pathways from various cell types converge to produce the EE-induced recovery from HX. We have also substantially revised the manuscript and added new data (including OPC-specific RNA-seq) that adds clarity to the mechanisms of action. Because these concerns are very similar to comments from Reviewer #1, please see our earlier responses, that we believe addresses each concern.*

Minor comments
(i) Why is the cellular response and outcome of EE different between TRAP mice and the CNP-EGFP mice (see Figure 1 vs Supplementary Figure 9). Moreover, are the EE-HX TRAP mice showing better outcomes than the normoxia control on the inclined beam test (Figure 9 F,G)?

- *We greatly appreciate this comment as it revealed a mistake in calculating the volume for these cell densities. We have corrected this mistake, nevertheless, slight strain-specific differences exist in OL lineage cellular responses after EE. These differences might be explained by EGFP expression in the CNP-EGFP mice. For quantifications in CNP-EGFP mice, expression of EGFP was a prerequisite for counting any OL lineage cells co-labeled with CC1, or NG2. This counting method yielded lower cell counts overall compared to the method used for TRAP mice, that didn't account for GFP expression. Therefore, it is possible that some CC1-expressing OLs decrease expression of EGFP in normally developing CNP-EGFP mice.*

- *As for the inclined beam data, there are no significant differences between NX and HX-EE mice on the 2-cm or 1-cm beam (Supp. 11f-g; p = 0.7 on 2-cm & p = 0.6 on 1-cm). This is also true for inclined beam testing on the PDGFRα-TRAP mice.*

(ii). In Figure 4D,C,E, is the difference between normoxia and normoxia EE significant? It seems that normoxia EE has fewer foot slips and is quicker than normoxia. What is the p value? Could it be that there is an increase in myelination with EE exposure? This is perhaps why there is such a great variance in Figure 2D,E (it might be better to quantify the proportion of myelinated axons per unmyelinated axons rather than the field of view).

- *In the inclined beam test (now Figure 3), there are no significant differences in foot slips between normoxic standard and enriched groups (p = 0.96 on 2-cm, p = 0.3 on 1-cm). However, we previously overlooked the fact that NX-EE mice traversed the beam faster than controls (p = 0.02 on 2-cm, p = 0.04 on 1-cm). It is possible that a slight increase in myelination is responsible for this change. Furthermore, the only significant change in OL lineage cell density between NX and NX-EE groups was in proliferating OPCs in the "delayed and extended" paradigm that was added to revised version of the manuscript (Page 11). These two findings suggest that long periods of enrichment may have effects on normally developing mice, but that these effects are modest and age-dependent.*

(iii) Representative images for barographs would be useful for interpreting the data in the manuscript.

- *We have added a number of representative images for quantifications throughout the manuscript.*

(iv) Figure 2 is missing information regarding which white matter tract is being quantified and where is it being quantified. Moreover, why is there such a great variance in the normox EE group and what is the cause of that? Are exactly the same locations within the white matter tact being compared?

- *EM analyses were conducted in the corpus callosum for all groups. This information was added to the text and figure legend (now Figure 1). It is possible that the myelination variance in NX-EE mice is due to modest EE-induced increases in myelination (as is discussed above).*

(v) On page 5, line 181, define "smaller." Quantify the difference and indicate whether it is significant or not. More generally, when comparing data throughout the manuscript, please give the average numbers and s.e.m., or the fold increase/decrease and/or p-value.

- *Upon revision, we have replaced this type of language with more quantitative language when describing differences in data. Mean and s.e.m or p-values have been added throughout the manuscript when describing significant differences.*

(vi) The resolution of the axis labels on bar graphs is poor. Labels and numbers are illegible. Please adjust.

- *All figures have been remade with high resolution images and charts.*

(vii) The referencing is not consistent. It needs to be made uniform.

- *Referencing inconsistencies have been corrected.*

REVIEWERS' COMMENTS:

Reviewer #1 (Remarks to the Author):

I thanks the authors for their careful response to my review and the addition of new data to address the concerns raised. I am not satisfied that this manuscript details novel findings that will be of significant interest to the research community, and I have no further issues to raise.

Reviewer #2 (Remarks to the Author):

This is a resubmission of a manuscript by Forbes et al. that focuses on the impact of environmental enrichment following perinatal brain injury. This study was extensive in its first submission and they now provide important new data supporting their conclusions that environmental enrichment improves recovery both at the cellular level and the behavioral level following perinatal hypoxia, a model that they have studied extensively. As before this is well written and expansive in the approaches they have taken to analyze these animals; they study the cellular, molecular and behavioral impact of environmental enrichment and present a full picture of its impact following hypoxic damage.
In general, the authors have addressed the issues in the earlier critique. This is an excellent study.
Of particular note: They have some very intriguing observations in these studies. Interestingly, despite high myelin protein expression after hypoxia, there is much less myelin, and they now show that there is a dramatic increase in the number of premyelinating oligodendrocytes, which presumably cannot complete their differentiation in that context. By contrast, after environmental enrichment, there is more myelin and reduced numbers of the premyelinating oligodendrocytes. Clearly following hypoxia there is a block in late oligodendrocyte differentiation and this block is alleviated by enrichment. This is an important observation and will have an impact in the field.
Their new data on the impact of this model on other cells and axons provides additional insight into damage and recovery in this model.
Further important data come from the BacTRAP studies. For example, they see that in animals without enrichment, the differences in translated RNAs in oligodendrocytes are completely eliminated by P45 despite the dramatic reduction in myelin in the hypoxia mice. As they conclude, this is important as it further supports that conclusion that their earlier work suggested, i.e., that intervention must occur at a critical window.
One editorial issue is the fact that at least in the PDF, the order of the figures is mixed up. They are as listed in the Nat Comm website, but not in the order of the figures in the manuscript. This made assessing the data more complicated. Also, the supplemental tables were mislabeled as table, making them hard to find.
Wendy Macklin

Reviewer #3 (Remarks to the Author):

The manuscript has greatly improved since last submission and the authors have address all my comments and concerns to a satisfactory level. - I have no further comments

Response to referees:

The authors greatly appreciate the careful and constructive comments that all referees have provided throughout the course of this peer review process. We are happy to have adequately addressed all referee concerns and look forward to the revised manuscript being accepted and published.

REVIEWERS' COMMENTS:

Reviewer #1 (Remarks to the Author):

I thank the authors for their careful response to my review and the addition of new data to address the concerns raised. I am now satisfied that this manuscript details novel findings that will be of significant interest to the research community, and I have no further issues to raise.

We are happy to have addressed all of your concerns, and are pleased that you find our results to be interesting to the research community.

Reviewer #2 (Remarks to the Author):

This is a resubmission of a manuscript by Forbes et al. that focuses on the impact of environmental enrichment following perinatal brain injury. This study was extensive in its first submission and they now provide important new data supporting their conclusions that environmental enrichment improves recovery both at the cellular level and the behavioral level following perinatal hypoxia, a model that they have studied extensively. As before this is well written and expansive in the approaches they have taken to analyze these animals; they study the cellular, molecular and behavioral impact of environmental enrichment and present a full picture of its impact following hypoxic damage.
In general, the authors have addressed the issues in the earlier critique. This is an excellent study.
Of particular note: They have some very intriguing observations in these studies. Interestingly, despite high myelin protein expression after hypoxia, there is much less myelin, and they now show that there is a dramatic increase in the number of premyelinating oligodendrocytes, which presumably cannot complete their differentiation in that context. By contrast, after environmental enrichment, there is more myelin and reduced numbers of the premyelinating oligodendrocytes. Clearly following hypoxia there is a block in late oligodendrocyte differentiation and this block is alleviated by enrichment. This is an important observation and will have an impact in the field. Their new data on the impact of this model on other cells and axons provides additional insight into damage and recovery in this model.
Further important data come from the BacTRAP studies. For example, they see that in animals without enrichment, the differences in translated RNAs in oligodendrocytes are completely eliminated by P45 despite the dramatic reduction in myelin in the hypoxia mice. As they

conclude, this is important as it further supports that conclusion that their earlier work suggested, i.e., that intervention must occur at a critical window.
One editorial issue is the fact that at least in the PDF, the order of the figures is mixed up. They are as listed in the Nat Comm website, but not in the order of the figures in the manuscript. This made assessing the data more complicated. Also, the supplemental tables were mislabeled as table, making them hard to find.
Wendy Macklin

We greatly appreciate your kind remarks. In response to your editorial issue, we have diligently ensured that all figures are uploaded in the correct order for this final submission. Additionally, we have appropriately labeled all supplemental tables as "supplementary data" at the request of the editors.

Reviewer #3 (Remarks to the Author):

The manuscript has greatly improved since last submission and the authors have addressed all my comments and concerns to a satisfactory level. - I have no further comments

We greatly appreciate your previous comments and concerns, and are pleased to have addressed them all to your satisfaction.
